# Drought stress tolerance mechanisms and their potential common indicators to salinity, insights from the wild watermelon *(Citrullus lanatus):* A review

**DOI:** 10.3389/fpls.2022.1074395

**Published:** 2023-02-06

**Authors:** Goitseone Malambane, Kelebogile Madumane, Lesego T. Sewelo, Utlwang Batlang

**Affiliations:** Department of Crop and Soil Sciences, Botswana University of Agriculture and Natural Resources, Gaborone, Botswana

**Keywords:** climate change, root growth, stomatal closure, chloroplast-APX, citrulline, DRIP1, RAN GTPase, type-2 metallothionein (MT)

## Abstract

Climate change has escalated the effect of drought on crop production as it has negatively altered the environmental condition. Wild watermelon grows abundantly in the Kgalagadi desert even though the environment is characterized by minimal rainfall, high temperatures and intense sunshine during growing season. This area is also characterized by sandy soils with low water holding capacity, thus bringing about drought stress. Drought stress affects crop productivity through its effects on development and physiological functions as dictated by molecular responses. Not only one or two physiological process or genes are responsible for drought tolerance, but a combination of various factors do work together to aid crop tolerance mechanism. Various studies have shown that wild watermelon possess superior qualities that aid its survival in unfavorable conditions. These mechanisms include resilient root growth, timely stomatal closure, chlorophyll fluorescence quenching under water deficit as key physiological responses. At biochemical and molecular level, the crop responds through citrulline accumulation and expression of genes associated with drought tolerance in this species and other plants. Previous salinity stress studies involving other plants have identified citrulline accumulation and expression of some of these genes (chloroplast APX, Type-2 metallothionein), to be associated with tolerance. Emerging evidence indicates that the upstream of functional genes are the transcription factor that regulates drought and salinity stress responses as well as adaptation. In this review we discuss the drought tolerance mechanisms in watermelons and some of its common indicators to salinity at physiological, biochemical and molecular level.

## Introduction

Climate change, which is characterized by high temperatures, erratic and unreliable rainfalls as well as an increase of desert margins has become a common phenomenon in the arid and semi-arid regions of the world. These factors and their interactions bring about undesirable environmental conditions that affect agriculture and most importantly crop productivity. Drought and salinity stress has been attributed to a combination of several factors but generally it is notable when there is a reduction of available water in the soil, and atmospheric conditions that cause loss of water by transpiration for a period of time (Jaleel et al., 2009; Takahashi et al., 2020). Drought and salinity affects crops from morphological to molecular level in varying levels, these effects can be observed at any phenological stage (Farooq et al., 2008; Sheoran et al., 2022). Crop productivity is highly affected by drought and salinity stress reasons being that the ability of plants in utilization of light energy to carry out photosynthesis is also highly influenced by accessibility to moisture. One of the physiological processes that are highly affected by these abiotic stresses is the photosynthesis which is essential in both plant growth, development, and productivity.

Climate model predictions show that the adverse effects such as drought are not going to soften up but rather get worse over a period of time, with the sub-Saharan region proving to be highly affected (Walter et al., 2011; Brown et al., 2012) thus becoming important to unravel the morphology, physiology and genetic makeup of crops from areas experiencing natural drought (Hussain et al., 2015). A case in point is the xerophyte wild water melon *(Citrullus lanatus)*, found in the Kalahari desert of southern Africa, where it is known to withstand extended periods compared to other C3 species. Drought stress tolerance occurs in almost all plants, but its extent varies from species to species and even within similar species great variation can be observed (Niinemets, 2015; Gorim and Vandenberg, 2017; Iseki et al., 2018). A significant difference in phenotypic and transcriptional regulation of genes during drought between wild plants and domesticated plants has been noted by Akashi et al. (2008); Iseki et al. (2018); Rosero et al. (2020). This then suggests that wild species have better mechanisms in dealing with abiotic as compared to their cultivated relatives (Ghorecha et al., 2017; Iseki et al., 2018), thus making them important to study and harness their tolerance mechanisms this also applies to cultivated sensitive crops. Therefore, this review aims at consolidating findings on mechanisms that aid one of the drought tolerant wild species (wild watermelon) that survive and produce well in the harsh Kalahari Desert conditions.

### The wild watermelon

Wild watermelon referred to as the wild cousin or the ancestor of the cultivated watermelon inhabits the Kalahari Desert which is in the western part of Botswana. The crop grows very well in this arid and hostile environments, however unsuitable for the cultivated watermelon, under above normal temperature and low rainfall. The soils are mostly sandy with low water holding capacity and less plant available nutrient required for growth. According to Gibson (1996) and Yokota et al. (2002), annual precipitation is about 200 mm, restricted to spring and summer during which annual plants thrive, but get exposed and suffer severe drought which is mostly survived by the wild watermelon.

Unlike its cultivated cousin, the fruits of wild watermelon are less palatable, not sweet, and the internal color is mostly white to creamy color and at times yellowish. The mature fruit has a similar size as the cultivated watermelon. However, a review by Mtumtum (2012) reported wild watermelons are edible and used in different forms, its leaves, flowers, and young fruit can be cooked as green vegetables. The seeds can be roasted and consumed as they are considered a delicacy. It contains relatively the same amount of water as cultivated watermelon; thus, it has become an important crop of the Kalahari Desert for it is a valuable source of water for the indigenous people and their livestock (Zulu and Modi, 2010). It is also an important part of cropping systems as the crop has been found to reduce the frequency of weeding and production costs by acting as a live mulch when grown as minor with other crops.

The crop survival in these extreme conditions of the desert suggests it to be drought tolerant, thus making it an important crop to study drought response mechanisms. Several studies on this matter relative to the mechanisms in aiding crop drought tolerance have been conducted. Further, the partial sequencing and isolation, characterization, as well as documentation for some of the important genes has been done and the information is available to be used in further studies. Furthermore, the species can be used as a model to elucidate the function of genes implicated in drought and salinity stress responses ultimately leading to enhancement of stress tolerance in cucurbit crops through genetic manipulation. Most importantly, the wild watermelon is an adaptable crop for addressing food security in the face of climate change.

## Morpho-physiological responses to environmental stress

### Resilient root growth under water deficit

Roots are a very important part of a plant during its growth and adaptation to stress especially the moisture deficit stress. Firstly, they are the anchors for every plant; and they also play an important role and are responsible for the nutrient, water uptake from the soil and sensing drought stress. The root growth evolution influenced by adaptation to the local environment is closely related with the plant phenotype, growth medium properties and the availability of moisture, temperature, and nutrients of the medium (Bao et al., 2014). The type of root varies with the crop species and its growth, development and intensity is influenced by environmental information (Malamy, 2005). The water absorption of various plants under environmental stress is influenced by various root features like the primary root length, and number of lateral roots. In the case of the wild watermelon, under drought stress the plant expresses it xerophytic characteristic by extending its geophyte-type root system into deep ground water. However, even when the root extension is restricted by growing in a pot the plant could still withstand severe drought showing that its tolerance. (Yokota et al., 2002).

The root system has been known to have a great effect on how plants adapt and respond to environmental stress including drought and salinity (Wasaya et al., 2018; Comas et al., 2013; Karahara and Horie, 2021; An et al., 2003) and the special characters aiding the roots to help plants better adapt to moisture stress are plasticity (Lynch, 2007) and the plasticity is controlled by genetic components triggered by abiotic stress and holds a great potential in stabilizing productivity under suboptimal conditions (Zhu et al., 2011; Gifford et al., 2013).

Several molecular mechanisms have been suggested and confirmed to be responsible for root plasticity when tolerant crops are subjected to abiotic stresses. Several quantitative trait loci has been found to be responsible for the longitudinal and latitudinal growth of roots to access deeper and wider moisture in mitigation effects of salinity and drought stress. DEEPER ROOTING 1 (DRO1), SURFACE ROOTING (SRO1), AUXIN RESPONSE FACTOR 7 (ARF7), HIGH-AFFINITY K^+^ TRANSPORTERS (HKT1) and CYCLOPHILIN (CYP) have been documented to be responsible in developing deeper and lateral rooting in various crops like rice (Singh et al., 2021; Uga et al., 2013), maize (Feng et al., 2022) and Arabidopsis (Guseman et al., 2017) thus suggesting an active role in the plants tolerance and avoidance to abiotic stress. Several other factors like QTLs and like qRT9 (Li et al., 2015), qTLRN, qLLRN (Niones et al., 2015) transcription factors like NACs (Thao et al., 2013), MYB (Zhao P. et al., 2019), NFY Family (Yang et al., 2022), has been suggested to play a significant role in the root architecture of plants during drought stress. Further molecular mechanisms playing an active role in the root morphological characteristics of various plants are documented in **Table 1**.

**Table 1:** Molecular mechanisms involved in plant tolerance against salinity and drought stresses.

Name	Molecular class	Abiotic stress	Function/Plant trait	Plant species	Reference
DEEPER ROOTING 1 (DRO1)	QTL	Drought and salinity	Regulation of root architecture	*Triticum aestivum, oryza sativa*	Kulkarni et al. (2017)
SIMILAR TO RCD ONE (SRO1)	QTL	Drought and salinity	Seedling growth, regulates ROS and Cellular redox homeostasis	*Solanum lycopersicum*	Liu et al. (2014)
qRT9	QTL	Drought and salinity stress	Root Architecture (root thickness and length)	*Oryza sativa*	Li et al. (2015)
Total Lateral root Number (qTLRN), L-type Lateral Root Number (qLLRN)	QTL	Drought	Root architecture Lateral root growth	*Oryza sativa*	Niones et al. (2015)
(Auxin Response Factor) ARF	TF	Cold, Drought and salinity	Lateral root development, leaf expansion senescence, fruit development	*Elaeis guineensis*	Jin et al. (2022)
*Myeloblastosis* (MYB)	TF	Drought and salinity	Cuticle development, stomatal aperture, ABA signaling	Watermelon (*Citrullus lanatus L*.) *Arabidopsis thaliana*	Xu et al. (2018) Wang et al. (2021)
Nuclear factor Y (NFYs)	TF	Drought and salinity	Maintaining stable relative water content (RWC)	Watermelon (*Citrullus lanatus L*.) *Arachis Hypogaea L.*	Yang et al. (2017) Wan et al. (2021)
9-*cis*-epoxycarotenoid dioxygenase (NCED)	TF	Drought and salinity	Inhibiting Al+ toxicity, Stomatal aperture	Watermelon (*Citrullus lanatus L*.) *Citrus limonia*	Li et al. (2012) Gavassi et al. (2021)
NAM, ATAF and CUC (NAC families)	TF	Drought and salinity	Cell division, leaf senescence, formation of secondary wall	Watermelon (*Citrullus lanatus L*.) *Glycine max, Oryza sativa*	Song et al. (2020) Shao et al. (2015)
Basic leucine zipper motif (bZIP)	TF	Drought and salinity	Vascular development in roots, leaf and root development	*Oryza sativa*	Yang et al. (2019)
basic Helix-Loop-helix (bHLH)	TF	Drought and salinity	Light signaling transduction, Plant growth, Metabolite biosynthesis	Melon (*Cucumis melo*) *Myrothamnus flabellifolius Welw, Populus euphratica Olivier*	Tan et al. (2021) Guo et al. (2021)
WRKY	TF	Drought and salinity	Leaf senescence, development, and secondary metabolites synthesis	Watermelon (*Citrullus lanatus L*.) Sweet potato *(Ipomoea batatas)* and Rice *(Oryza sativa)*	Yang et al. (2018) Jiang et al. (2017)
Dehydration Responsive Element Binding (DREB)	TF	Drought and salinity	Signal transduction	*Arabidopsis thaliana* and soybean (*Glycine* *max*)	Niu et al. (2020)
Abscisic Stress induced Ripening (ASR)	Functional Protein	Drought and salinity	Modulating stomatal aperture, fruit ripening	*Oryza sativa*	Park et al. (2020)
High-affinity K+ (HKT)	Functional Protein	Salinity	Na+ removal from the xylem	Pumpkin (*Cucurbita moschata)* *Glycine max*, *Sorghum bicolor*	Sun et al. (2018) Li et al. (2019)
Cyclophilin (CYP)	Functional Protein	Drought and salinity	Cellular signaling, maintaining ion homeostasis, protein degradation and apoptosis	*Oryza sativa, Brassica rapa*	Olejnik et al. (2021)
Drought-Induced Peptide (DRIP)	Functional Protein	Drought and salinity	Accumulation of osmoprotectants	Wild watermelon (*Citrullus lanatus L*.)	Yokota et al. (2002)
CLAVATA3/ Embryo Surrounding region (CLE)	Functional Protein	Drought	Stomatal aperture	*Arabidopsis thaliana*	Zhang et al. (2019)
C-TERMINALLY ENCODED PEPTIDE (CEP)	Functional Protein	Drought and salinity	Tunes auxin signaling, lateral root growth	*Arabidopsis thaliana*	Smith et al. (2020)
Heat Shock Proteins (HSPs)	Functional Protein	Drought and salinity	Protect the Photosystems and thylakoid membrane	Wild watermelon (*Citrullus lanatus L*.) Rice *(Oryza sativa)* and Wheat (*Triticum aestivum*)	Akashi et al. (2011) Mishra et al. (2018)
Late Embryogenesis Abundant (LEA)	Functional Protein	Drought and salinity	Stabilizing water homeostasis and ROS Scavenger	Wild watermelon (*Citrullus lanatus L*.) Potato (*Solanum tuberosum*	Akashi et al. (2008) Chen et al. (2019)
Dehydrins	Functional Proteins	Drought and salinity	Chaperons, chelators, free radical scavenging	Watermelon (*Citrullus lanatus L*.) *Arabidopsis thaliana* and Rice *(Oryza sativa)*	Lee et al. (2017) Tiwari and Chakrabarty (2021)
GTPase	Functional Proteins	Drought and salinity	Root hair development, pollen growth, hormonal signal transmission	Wild watermelon (*Citrullus lanatus L*.) Wheat (*Triticum aestivum*)	Akashi et al. (2016a) Choudhury et al. (2021)
Trehalose-6-phosphate synthase (TPS)	Functional Protein	Drought and salinity	ROS Scavenging, regulating Intracellular K+ /Na+ balance	Watermelon (*Citrullus lanatus*)	Yuan et al. (2022a) Yuan et al. (2022b)
Aquaporins	Functional Proteins	Drought and salinity	Regulated water transport and stomatal aperture	Watermelon (*Citrullus lanatus*)	Raturi et al. (2022)
Thaumatin-like protein (TLP)	Functional Protein	Drought and salinity	Immune response, stomatal aperture	Watermelon (*Citrullus lanatus*)	Ram et al. (2022)
Metallothionein (MT)	Functional Proteins	Drought and salinity	Root growth, transpiration rate, stomatal aperture, accumulation of compatible solutes	Wild watermelon (*Citrullus lanatus L*.) *Arabidopsis thaliana*	Akashi et al. (2004) Patankar et al. (2019)

Some mechanisms have already been documented to play an important role in wild watermelon tolerance mechanisms while the rest are potential molecular mechanisms behind the morphological and physiological tolerance mechanisms in wild watermelon. Quantitative trait Loci, (QTL); Transcription Factors, (TF).

**Figure 1:**
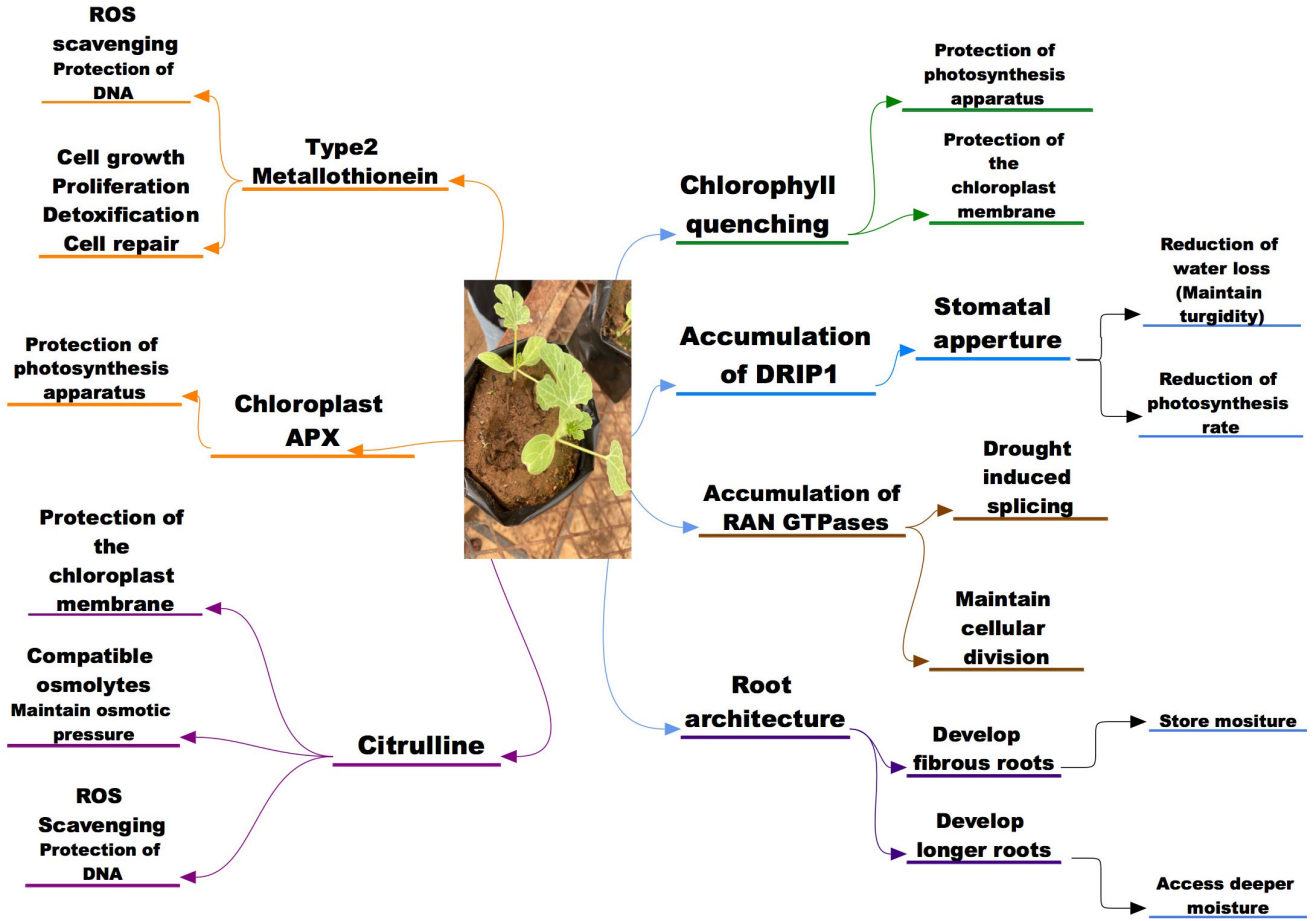
A schematic representation of the morphological, physiological and molecular potential indicators to drought and salinity stress tolerance in wild watermelon.

In wild water melons, the optimization of the root architecture to maximize use of deep water has been noted as an advantageous trait, as growing in drought stress condition a vigorous root growth was observed (Akashi et al., 2016a). In studying the root structure of wild watermelon Yoshimura et al. (2008) found out that when exposed to moisture stress, the root development is significantly an indication of triggered drought avoidance mechanisms aiding access to moisture in deeper soils (**Figure 1**). The good rooting pattern, densities and hydraulic conductance of the wild watermelon has been attributed to superior ability to access deeper water thus aiding its drought tolerance mechanisms (Smith, 2006), while the tap root and the semi-taproot that grows deeper as well as the root system that is spreading has also shown to contribute immensely to the tolerance mechanisms of wild watermelon (Condon and Hall, 1997). A proteome analysis on the root of wild watermelon exposed to drought stress revealed increased expressions levels of proteins associated with root morphogenesis and primary metabolism and these correlated with the enhanced root development (Yoshimura et al., 2008). These traits of the wild watermelon points to established drought tolerance mechanisms aiding the crop to survive under the harsh environmental conditions.

To study the root roles in the tolerance mechanism of the wild watermelon, Kajikawa et al. (2010) further developed a transgenic root hair system to better understand the root physiology and drought stress and the effects after exposure to stress, substantial growth under stressful osmotic conditions as compared to the cultivated watermelon which had their roots growth highly inhibited has since been observed. The role of the resilient wild watermelon root was also observed when Seymen et al. (2021) used wild watermelon rootstock in grafting of cultivated watermelon. When exposed to moisture stress conditions, the grafted plant showed superior tolerance to the stressful conditions as compared to un-grafted plants and that has been attributed to the role of the rootstock’s roots.

The root morphology and physiology also aids plants to sustainable growth under salt stress; importantly the plasticity of roots have been noted to be instrumental in aiding plants to tolerate salinity (Arif et al., 2019). It has been noted that the plasticity assists plants by preventing accumulation of salts in roots thus allowing normal uptake of moisture from the soils (Schleiff and Muscolo, 2011). Li et al. (2014) also noted that changes in root morphology as a critical factor for plants in tolerating salt stress. This was ascertained as a higher root biomass and root length was observed in *Suaeda salsa* (Wang et al., 2021), *Robinia pseudoacacia* (Mao et al., 2016 *) and Jerusalem artichoke* (Yang et al., 2016) as a salinity tolerance mechanisms. The rapid growth of root hairs has been positively identified to contribute to the increase in root densities thus aiding the tolerance mechanisms in plants exposed to saline soils (Arif et al., 2019). This phenomenon is only observed in salt tolerant plants as susceptible species have shown a contrasting response when exposed to saline conditions. Chang et al. (2019) observed that when three rice plants were exposed to saline soils, they recorded a significant reduction in root biomass and length as the intensity on the salinity increased. Several other salinity susceptible plant species also showed similar reduced root morphology when exposed to saline conditions (Ashraf et al., 2005; Arif et al., 2016). Cultivated watermelon has been recorded to be salt sensitive and thus attempts to improve the crop has primarily focused on the use of salt tolerant roots stocks to graft the crop. Watermelon showed great resistance to salinity when grafted to the salt tolerant species which develop a well-established root biomass that aided the grafted plants to accumulate less salt ions thus water uptake was not limited (Yan et al., 2018: Zhu et al., 2018; Kuşvuran et al., 2021). This shows that improvement into the root system (be it morphological, physiological or molecular) will enhance the salinity tolerance of cultivated watermelon. With the wild watermelon showing a good establishment of root plasticity under other abiotic stress, it thus presents a potential model to understand the roots response of this and related species in stress tolerance studies.

### Timely stomatal closure response to drought stress

Under normal circumstances gaseous exchange in plants is facilitated by the stomatal aperture; whereby the opening allows for CO_2_ to be taken in for the process of photosynthesis while O_2_ is taken out as a by-product. Stomatal opening responds to several environmental and physiological factors, such as; light, leaf-to-air vapor pressure deficit, drought and salinity stress (Farquhar and Sharkey, 1982). Plant responses to salinity and drought are often similar. The first phase of salinity stress is the osmotic effect, which is quite similar to that of drought stress (Yildiz et al., 2020). The presence of excess salts in soil causes osmotic stress formation by limiting water availability of plant with roots. In a similar way, osmotic stress is induced by low water potential that results from an actual lack of water in an environment, due to low precipitation (drought). In both cases plants cannot uptake sufficient water for normal growth and development and common stress related signal transduction pathways are activated.

Closure of stomata has been noted as a first response of plant to osmotic stress (Munns and Tester, 2008) and this is highly dependent on the plant species, where tolerant species will have a mechanism of controlling the stomata to allow partial gaseous exchange to facilitate the photosynthesis process (Pirasteh-Anosheh et al., 2016). Under drought stress where soil moisture is limited and/or high atmospheric evaporative demand, partial or complete stomatal closure allows plants to maintain a favorable water balance while limiting the carbon gain. Salinity-generating stress also results in stomatal regulation, an important strategy that enables plants to cope with NaCl-induced osmotic and ionic stresses (Orzechowska et al., 2021). Plants exposed to salinity close their stomata to protect against water loss. The opening is limited by the soil and atmospheric moisture content because during moisture deficit the stomata tends to reduce their opening as an established form of first line of defense mechanism (Mansfield and Atkinson, 1990; Cornic and Massacci, 1996; Singh and Reddy, 2011; Hou et al., 2016). In doing so, excessive loss of leaf moisture and desiccation is avoided, thus protecting the cell structure and metabolisms. Stomatal closure plays an important role in protecting the most sensitive photosynthesis apparatus of the plant thus enabling the plant to survive/regenerate post the stress period (Martin-StPaul et al., 2017). Therefore, when the stomatal and non-stomatal limitations to photosynthesis are compared, the stomatal closure outweighs the later in plant growth and survival under stress conditions (Escalona et al., 2000; Tissue et al., 2005).

The response time of stomata during the critical period is very important in the survival of plants during drought and salinity stress, the quicker the plants respond, the better position they are in avoiding stress effects. This has been associated with the ability to withstand drought in *phaseolus* (Markhart, 1985) cowpea (Laffray and Louguet, 1990), sorghum (Tsuji et al., 2013), soybean (Hossain et al., 2015) and woody species (Yan et al., 2017); salinity in maize (Neto et al., 2004), Cotton (Janagoudar, 2007), sorghum (Duarado et al., 2022), *Eruca sativa* (Hniličková et al., 2017). This was attributed to stomatal closure as a form of salinity stress coping mechanism. The stomatal response can take several forms depending on the species and drought tolerance. Stomata can completely be closed under severe drought stress, and this is closely dependent on plant species, as tolerant species tend to control status of their stomata to allow carbon fixation and photosynthesis as well as improving water use efficiency (Laffray and Louguet, 1990; Pirasteh-Anosheh et al., 2016).

Stomatal aperture is highly regulated by the accumulation of the ABA. Several molecular mechanisms were found to play in the ABA-mediated stomatal aperture by triggering the ABA synthesis under drought stress (Daszkowska-Golec and Szarejko, 2013). Stress and ripening (ASR) gene family has been documented to play a significant role in the opening and closing of stomatas during abiotic stresses (Li et al., 2017) with several studies confirming the role in stomatal aperture. Other gene families like NCED (Sussmilch et al., 2017; Gavassi et al., 2021) and RAB have also been documented to play an important part in stomatal aperture during environmental stresses. Transcription factors like MYB (Oh et al., 2011; Simeoni et al., 2022) and Cyclophilin CYP (Liu et al., 2021; Olejnik et al., 2021), NAC (Du et al., 2014; Negi et al., 2018) and NFY (Guochao et al., 2017; Yu et al., 2021) families have been found to play a significant role in the ABA induced stomatal aperture

In comparison with cultivated watermelon, the wild watermelon has shown a different response pattern in stomatal closure when exposed to moisture deficit. The wild watermelon showed a rather quick stomatal response as observed by a reduced internal CO_2_ and dropped to almost zero in the earlier stages of moisture deficit; a same observation was made when the wild watermelon was compared to other crops in the citrullus family (Nanasato et al., 2010; Sanda et al., 2011; Mo et al., 2015; Malambane, 2018a). The stomatal closure of the wild watermelon response was observed within 3 days of moisture stress induction while the cultivated watermelon and cucumber took longer period for the internal CO_2_ levels to drop. The wild watermelon internal CO_2_ dropped to almost zero within 5 days post stress and was quicker as compared to the cultivated watermelon that took longer for the internal CO_2_ to approach zero level. The timely stomatal closure of the wild watermelon results in lower internal CO_2_ which then results in significantly reduction of the photosynthesis activity, and this can be thought to contribute to the survival of the plant as the energy is focused mostly in maintaining the plant under drought stress (**Figure 1**). The stomatal response of the wild watermelon to drought has shown similar trends as stomatal response to salinity in other crop species like melon, bottle gourd, pumpkins, luffa studied by Modarelli et al., 2020) and Arabidopsis thaliana (Orzechowska et al., 2021) thus the timely stomatal closure can be considered an important tolerance mechanisms for the wild watermelon during both drought and possibly for salinity stress too. However, in terms of salinity tolerance, precise physiological mechanisms need to be established in addition to safe guarding photosynthetic machinery through stomatal closure.

### Protection of photosynthesis apparatus through chlorophyll fluorescence quenching

Stomatal closure as a response to abiotic stress usually results in a decrease in CO_2_ supply to mesophyll cells and subsequently to the photosynthesis process. When drought is severe, this will be lead to a decrease in the rate of ATP and NADPH consumption for CO_2_ assimilation (Baker and Rosenquist, 2004). In most sensitive plant species this would result in decreases in the rate of linear electron transport and consequently in operating efficiency of the most sensitive photosystem II (Φ_PSII_) or the efficiency at which light absorbed by PSII antennae is used for reduction (QA reduction). In a similar manner, severe salt stress decreases pigment content and activity of photosynthetic electron transport (ΦPSII, qP), inhibits conversion (Fv/Fm) of light energy, and destroys cell membrane structure in plants (Jia et al., 2019). Chlorophyll (Chl) is an important pigment that reflects plant photosynthetic capacity and decrease in its content may be attributed to increased degradation and inhibited synthesis of the pigment. The loss of Chl is usually accompanied by inactivation of photochemical reactions, especially those mediated by PSII in plants exposed to salt stress and all this impact photosynthesis. Under drought and salinity stress, excess light energy absorbed by chlorophyll can cause irreversible damaged to PSII lead to cell death if not safely dissipated (quenched). This quenching acts as a protective mechanism that prevents the formation and accumulation of reactive oxygen species (ROS) induced by excessive reduction of the primary acceptor of PSII (Demmig-Adams and Adams, 2006; Zivcak et al., 2014). Quenching analysis allows for the separation of the contributions of photochemical and non-photochemical processes in the quenching of variable fluorescence, by inducing a temporary closure of all PSII reaction centers by a strong saturating light pulse (Baker, 2008). The decrease in fluorescence due to photochemistry is named photochemical quenching and the most useful parameter derived from quenching analysis is the measure of the efficiency of PSII (Φ_PSII_) and quenching, qP indicates the proportion of open PSII reaction centers (Maxwell and Johnson, 2000) while the non-photochemical quenching (NPQ) represents the rapid and reversible thermal dissipation (heat loss) of absorbed light energy in the PSII antenna (Horton and Ruban, 2005). NPQ is believed to quench about 90% of the excitation energy in the PSII, has a mechanism that induce dehydration and this mechanism is thought to highly contribute to the high tolerance of some plants against severe dehydration caused by drought and salinity (Komura et al., 2010).

When comparing various genotypes of cotton under well-watered conditions, Zakhidov et al. (2016) found out that the stressed plants displayed higher values of Φ_PSII_ and most important in the drought tolerant lines the values were even higher by about 15% suggesting that chlorophyll fluorescence quenching efficiency is higher in tolerant species. When *Ginkgo biloba L.* seedlings were exposed to salinity stress, the results shows that maximum (Fv/Fm) and actual (ΦPSII) quantum yields of photosystem II (PSII) decreased gradually in the higher concentrations salt treatments and stability of the membrane system are greatly affected (Zhao H. et al., 2019) indicates the aggravation of the PSII reaction center at greater stress levels (Lu and Zhang, 2000; Percival, 2005), and this corresponds with diminished photosynthesis (Percival and Sheriffs, 2002; Kalaji et al., 2011). An analysis of whether chlorophyll fluorescence quenching performance was affected by the environment by Sanda et al. (2011); Malambane et al. (2021) showed that the wild watermelon NPQ and Φ_PSII_ values were higher when compared to the cultivated watermelon either in stressed or unstressed conditions in the two varying environments. This increase can be associated with the protection of the PSII from damage by the reduced rate of electron entry into the PSII. This suggests that the photochemical and non-photochemical quenching capacity of wild watermelon supersedes that of the cultivated relatives in any condition, thus suggesting a better response of the wild relative to drought stress. The superior quenching of the wild watermelon was associated with the photosynthesis pathway as Nanasato et al. (2010) observed that wild watermelon as a C_3_ plant performed better than the C_4_ plants under drought stress. This was further ascertained by Guidi et al. (2019) who concluded that C_4_ plants species are inferior when subjected to other abiotic stressors such as drought with respect to photo-inhibition. Even though the C_4_ plants have a good acclimation potential, it has been reported that in most cases they lag behind the C_3_ plants in acclimation responses (Sage and McKown, 2006) and one of the reasons attested to the quick acclimation of the C_3_ plants is their system which allows photosynthetic plasticity to be concentrated at the cellular level rather than at tissue level. In studying temperature acclimation and temperature adaptation (Yamori et al., 2014) observed that the C_3_ plants had a greater ability for temperature acclimation across broad range of temperatures as compared to the CAM and C_4_ plants. ATP synthase has been documented as an important bioenergetic engine for all organisms, however the role of these important enzymes in the response to environmental fluctuations has been under study and some studies have shown potential involvement in drought and salinity stress tolerance (Zhang et al., 2008; Michaletti et al., 2018; Liu et al., 2021). When exposed to drought stress, wild watermelon levels of showed a photosynthetic activity decrease by approximately 50% as compared to the control plants (Kohzuma et al., 2009). The decrease was then associated with the photoprotection brought about by rapid increase of the qE which then suggested that the expression of ATP Synthase is an important part in the plant’s acclimatization to environmental stress in wild watermelon (Hoshiyasu et al., 2013). When wild watermelon was exposed to high light under drought stress, the qE dramatically increase was observed by (Kohzuma et al., 2008) and this was attributed to the mechanisms that stabilizes the thylakoid pH. The protection mechanisms have further been explained in Dietz et al. (2001) where it was stated that this decrease in the ATPase subunits is important in over acidification of the thylakoid membrane thus protecting the photosynthesis apparatus.

Even though the protection of the photosystems by fluorescence quenching has been strongly suggested as one mechanisms for plants survival under drought stress, this can be suggested to be in response to light stress but less of moisture stress. Salinity stress imposes similar effects to plants as moisture stress because they all contribute to osmotic stress. But studies relating salinity stresses to PSII phytochemistry are conflicting and still inconclusive with some showing salinity inhibiting PSII activity while others showing PSII unaffected by the salinity stress. When comparing saline sensitive Arabidopsis to the tolerant Thellugiella, Stepien and Johnson, (2009) showed contrasting responses in the photosynthetic apparatus with Thellugiela inducing an alternative apparatus that aided it to tolerate salinity better that sensitive Arabidopsis. Exposure of the *Suaeda salsa* to saline environment showed no effect on any of the chlorophyll fluorescence parameters (Lu et al., 2003) this then suggests that in saline tolerant species there is no or limited changes in the PSII photochemistry thus suggesting the quenching mechanism to play a lesser important role in the plants salinity tolerance mechanism.

### Citrulline accumulation as an osmo-protectant

Citrulline is a non-protein amino acid first identified from the juice of watermelon, *Citrullus lanatus* and found to also occur in other cucurbitaceous fruits. However, it ubiquitous in animals, bacteria, fungi, and plants (Joshi and Fernie, 2017). Exposure of plants to abiotic stress disrupts the electron transport chains resulting in the accumulation of ROS, which can lead to cellular damage and in response citrulline and other amino acids are produced to act as more potent ROS-scavengers or antioxidants, which can protect DNA and metabolic enzymes from oxidative damage (Akashi et al., 2001; Verslues and Juenger, 2011). Citrulline is biosynthesized from arginine synthesis pathway *via* the nitric oxide synthase as signaling mechanisms with several enzymes are involved. Three key enzymes that are involved in the synthesis of citrulline are NO synthase (NOS), ornithine carbamoyltransferase (OCT), which produces Cit, and argininosuccinate synthetase (ASS), which converts it into argininosuccinate (Anwar et al., 2021).

Watermelons and other plants have been documented to accumulate citrulline in response to osmotic (Dasgan et al., 2009; Cao et al., 2017), salinity (Kuşvuran et al., 2013) and drought stress (Kawasaki et al., 2000; Garg et al., 2016; Khan et al., 2019; Song et al., 2020). In cultivated watermelon a study by Song et al. (2020) reported a rapid accumulation of citrulline and related metabolites in the vegetative tissues due to drought stress. The study concluded that the metabolic pathways associated with citrulline synthesis and catabolism is regulated in the vegetative tissues of watermelon and its functional significance during drought stress. In previous study, Akashi et al. (2016b) revealed that spatial and developmental patterns of citrulline accumulation in wild watermelon during drought were largely different from those of the antioxidant lycopene, total proteins, and soluble sugars (glucose, fructose, and sucrose); thus, suggesting the accumulation may be regulated in a different manner from other nutrients during development. Wild watermelon has been found to primarily accumulate citrulline, and then glutamate and arginine, in place of proline and glycine betaine (Kawasaki et al., 2000). When compared to other compatible solutes like mannitol, proline and glycine betaine in wild watermelon leaves, citrulline had a much higher hydroxyl radical scavenging activity (Akashi et al., 2001). Osmolyte compatibility results from the absence of its interactions with substrates and cofactors, and the nonperturbing or its favorable effects on macromolecular-solvent interactions. It is likely that citrulline as an osmolyte accumulates in cells under drought and salinity and balance the osmotic difference between the cell’s surroundings and the cytosol.

A much quicker response to salinity stress was observed in tolerant melons as compared to the sensitive ones where citrulline accumulation was observed 4 days earlier in tolerant species as compared to sensitive (Dasgan et al., 2009). In another study, Kusvuran et al. (2013) reported that citrulline is an important biochemical indicator in tolerance to salinity stress as salt tolerant melon genotypes accumulated more citrulline than the salt sensitive melon genotypes, suggesting that citrulline overproduction might be a consequence of adaptation to high saline and drought conditions. Despite the fact that citrulline accumulates in watermelon and other cucurbits in response to oxidative stress, (Akashi et al., 2001) reported that wild watermelon overproduced gamma-aminobutyric acid (GABA), proline and glutamine, not citrulline when it was subjected to saline conditions and the reasons for that metabolic salt response remains to be explained. However, citrulline was reported as the most efficient ROS scavenger compared to proline, mannitol and glycine betaine and effectively protecting DNA and metabolic enzymes from oxidative damage (**Figure 1**). Song et al. (2020) noted that the increased levels of citrulline protects the DNA cleavage and metabolic enzymes damage caused by ROS attacks, thus aiding the abiotic stress tolerance mechanisms of wild watermelon. Positive correlations have been found between citrulline accumulation and salinity drought stress tolerance in watermelons (Kusvuran et al., 2013). Salt tolerant melons (*Cucumis melo* L) genotypes have also been found to accumulate more citrulline in their leaves than sensitive (Dasgan et al., 2009). Furthermore, transgenic approaches have demonstrated a positive association between increased citrulline accumulation and drought and salt stress tolerance in Arabidopsis (Kalamaki et al., 2009a; Massange-Sánchez et al., 2016). Therefore, these accumulation and functionality of citrulline suggest it to be a key player in drought and potentially salinity tolerance mechanism in wild water melon and other crops.

### Protection of chloroplast through accumulation of ascorbate peroxidase

Reactive oxygen species (ROS) are produced in normal manner as a signaling mechanism during plant growth, but when they are exposed to unfavorable environmental conditions their production is increased. Under drought stress closure of stomata and consequent decrease in CO_2_ concentration in the leaf mesophyll results in the accumulation of NADPH in the chloroplasts. Under such conditions, O_2_ acts as an alternative electron acceptor resulting in the generation of ROS (Sminorff, 1993). High levels of ROS in plant cells are toxic to enzymes, proteins, lipids, and DNA resulting in death, thus it is important for plants to quench this toxic levels of ROS. Transcriptome studies have shown increased abundance of response genes to oxidative stress due to drought in maize (Kakumanu et al., 2012), Arabidopsis (Desikan et al., 2001; Baxter et al., 2007) Rice (Zhang et al., 2017a) and tobacco (Zhang et al., 2017b). In addition to the non-enzymatic, the mechanisms that plants use to quench these ROS are the enzymatic antioxidant which are capable of quenching the ROS and bringing them to tolerable levels.

One major important enzymatic antioxidant is the Ascorbate Peroxidase (APX) (EC 1.11.1.11) which belongs to the class I heme-peroxidase (Asada, 1992; Lazzarotto et al., 2011). The APX exists in various isoforms classified according to their subcellular locality and they are; soluble isoforms found in cytosol (cAPX), mitochondria (mitAPX) and chloroplast stroma (sAPX), while membrane-bound isoforms are found in microbody (including peroxisome and glyoxisome) (mAPX) and chloroplast thylakoids (tAPX) (Shigeoka et al., 2002; Caverzan et al., 2012). The sAPX and tAPX are collectively referred to as the chloroplast APX (chlAPX) as they are found in the leaf chloroplast membranes. Several studies have pointed out that APX plays a key role in various plant abiotic stress response and recovery post stress exposure (Fini et al., 2012; Kausar et al., 2012; Zarei et al., 2012). Of the different isoforms of APX, the chloroplast APX gene expression was shown to be stimulated earlier in the tolerant cowpea cultivars under drought (D’Arcy-Lameta et al., 2006), thus suggesting that the chloroplast APX (chlAPX) responds first during drought stress protection. One characteristic of the chlAPX is that it is extremely sensitive to H_2_O_2_ under low-level ascorbate (Miyake and Asada, 1996). In most plants the cAPX showed high accumulation during drought stress (Yoshimura et al., 1998), leading to suggestions that cAPX might be one of the initial targets of oxidative injuries in plant leaves under drought (Shikanai et al., 1998).

However, this tendency was in contrast in wild watermelon as chlAPX accumulation was higher compared to other isoforms during drought stress period while the change in cAPX was non-significant throughout the experimental period (Nanasato et al., 2010). The up-regulation of the chlAPX was also observed in the domesticated watermelon thus suggesting that the behavior is common for the *Citrullus* family. While this response of chlAPX in watermelon has not been conclusively correlated with drought tolerance, the pattern is similar to other factors that contribute to the drought tolerance in wild watermelon. Malambane et al. (2018b) further reported chlAPX 3 folds enzymatic activity as compared to that of cAPX cytosolic in wild watermelon under drought stress, thus confirming its importance during drought stress in wild watermelon. The accumulation pattern of the chloroplastic APX in wild watermelon when exposed to the moisture deficit suggests that it contributes to the tolerance mechanisms of the plants to drought stress by quenching the radicals thus protecting the photosynthesis apparatus of the plant (**Figure 1**). Nanasato et al. (2015) observed that the biochemical process that is involved in the *cyt b* 561-ascorbate oxidase redox chain was significantly fortified in wild watermelon during exposure to drought stress (moisture deficit and excess light). These results led to them proposing that this redox chain plays an important role in dissipating excess light thus aiding the crop to withstand the drought conditions.

Salinity stress creates ion imbalance and induces physiological drought like conditions by limiting the amount of water available to the plant, leading to lipid peroxidation and the production of ROS (Pandey et al., 2017). Under saline conditions, APX provides salinity tolerance at different levels to the affected plants (Sofo et al., 2015), thus APX is involved in the homeostasis of ascorbate, detoxification of H_2_O_2_, and the balancing of intracellular ROS messenger network (Diaz-Vivancos et al., 2013; Hernandez et al., 2001). Shalata et al. (2001) reported that during saline conditions, pea chloroplast APXs behaved differently, with sAPX increasing and tAPX decreasing gradually, while tAPX from tomato expressed in tobacco provided increased tolerance to salt and osmotic stress. In another study, Weisany et al. (2021) reported that APX transcripts in soybean increased due to salinity stress, while (Lin and Pu, 2010) documented that differential accumulation of APX transcripts with higher levels in tolerant genotypes were shown in sweet potato plants differing in their level of sensitivity to salt stress. The expression of cAPX, Mapx and chlAPX after exposure to 450mM NaCl in sweet potatoes plants was reported to be tissue specific and dependent on salt stress duration (Lin and Pu, 2010). Further, a cAPX from *Arabidopsis* in transgenic tobacco increased salt, drought, and PEG tolerance, and tomato plants over expressing pea cAPX were reported to be tolerant to salinity stress (Badawi et al., 2004; Wang et al., 2005). In comparison, over-expression of an APX from *Puccinellia tenuiflora* in Arabidopsis increased its tolerance to 175mM NaCl in addition to protection to lipid peroxidation, suggesting that APX provides salinity tolerance (Guan et al., 2015). Additionally, when analyzing for response of major antioxidant enzymes transcripts for different developmental stages in salt stressed rice, cAPX was up-regulated in 11-day-old seedlings, while in 6-week-old plants salt had no significant on this gene (Menezes-Benavente et al., 2004). The differences might be due to differences in cultivars, plant age and growth conditions (Tátrai et al., 2016).

## Molecular responses

### Expression of small signaling peptides and responsive proteins

**Figure 2:**
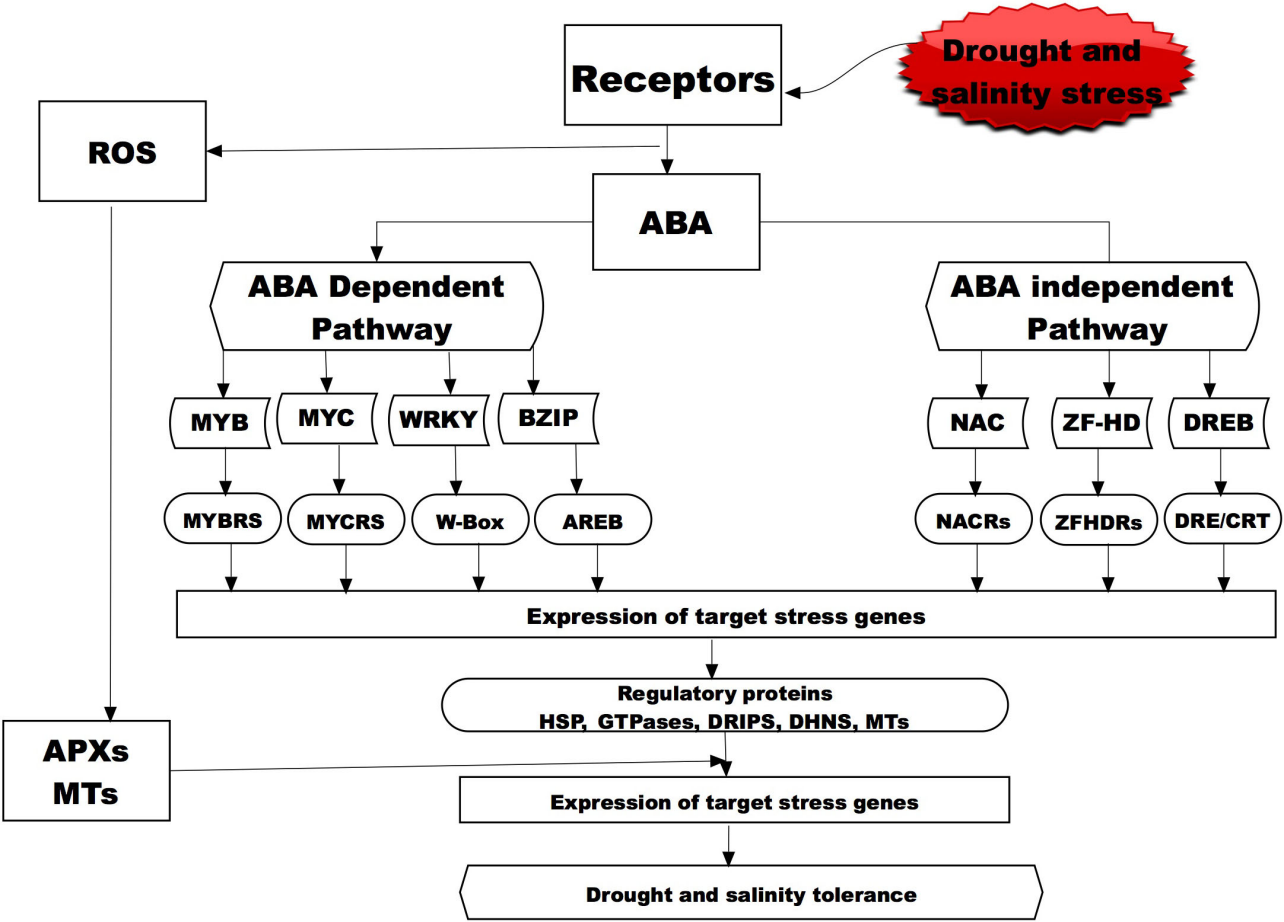
A representative diagram showing the molecular signaling for drought and salinity stress in plant through the ABA-dependent and independent pathways. Bolded and broken boxes show the molecular mechanisms that have been studied and presented in this review for the wild water melon drought tolerance and potential salinity tolerance indicators. Abscisic acid, (ABA; Myeloblastosis oncogene, (MYB); Myelocytomatosis oncogene, (MYC); WRKY Basic Leucine Zipper, (BZIP); NAC (NAM, ATAF and CUC) Zinc-finger homeodomain, (ZF-HD); Dehydration responsive element binding proteins, (DREB); MYB recognition site, (MYBRs); MYC recognition site, (MYCRs); ABA-responsive element binding protein, (AREB); NAC recognition site, (NARC); ZFHDR, cis-acting element/C-repeat DRE/CRT; Heat shock Proteins, (HSP); Drought induced proteins, (DRIP); ascorbate peroxidase, (APX); metallothionein, (MT); DHN, (Dehydrins).

Small signaling peptides play important roles in coordinating the intercellular communication in multicellular organisms (Yokota et al., 2002; Wang et al., 2016). These peptides have been found to play a role in wide-range of plant developmental and physiological processes (Feirs et al., 2005; Yaginuma et al., 2011; Song et al., 2013; Czyzewicz et al., 2015) and response against unfavorable stimuli (Lay and Anderson, 2005; Chen et al., 2019). The most common peptides associated with regulating against moisture stress are DRIP, CLE, IDA, PSK, CEP5 peptides while those regulating salinity responses are CAPE, RALF, AtPEP3 Peptides (Kim et al., 2021), and when exposed to osmotic stress these peptides have shown a similar accumulation pattern as a response mechanism of crops (**Figure 2**). Association of the response pattern of these peptides has been associated with several response factors of plants ABA accumulation and stomatal closure (McLachlan et al., 2018; Takahashi et al., 2018; Qu et al., 2019) in response to osmotic stress caused by either moisture or salinity. For instance, an AtMYB44 transcription factor involved in ABA-dependent and independent signaling pathways regulate stress adaptation and confer plant tolerance to salt stress (Nguyen and Cheong, 2018). Other transcription factors (AP2-EREBP, bZIP, bHLH, MYB, NAC, OFP, TCP, and WRKY) that are known to be acting in a similar manner through ABA signaling were recently determined by an RNA-Seq transcriptome analysis to be responsive and potentially contributing to salinity tolerance in water melons (Zhu et al., 2022). A transcriptome analysis of watermelon roots under osmotic stress revealed several response genes like Treahalose phosphate synthase (TPS) and Treahalose phosphate phosphatases (TPP) genes which were upregulated hence being suggested to aid plants tolerance mechanisms (Yang et al., 2016). In another transcriptome analysis study by Song et al., 2020, several differentially expressed genes were documented under osmotic and salinity stress with members of the ERF, WRKY, NAC, bHLH and MYB been over-represented suggesting their high active role in the tolerance mechanisms of watermelon seedlings. Yuan et al. (2022b) observed that genes involved plant hormone signal transduction, carbohydrate biosynthesis pathways were highly active in facilitating the tolerance mechanisms of watermelon seedlings against salinity stress. In another study by Zhang et al. (2020) it was found that overexpression of a MYB (SlMYB102) transcription factor in tomato *(Solanum Lycopersicon)* conferred salinity tolerance through increased upregulation of salinity stress related genes, increased the K+/Na+ ratio and the activity of active oxygen scavenging enzymes (SOD, POD, CAT, APX) and accumulation of the non-enzymatic antioxidants (ASA and GSH). These findings suggest that the response of watermelon to salt stress could be through a complex gene regulatory network, and MYB transcription factors may play an important role in salt tolerance by regulating downstream corresponding genes and the antioxidant systems.

One group of peptides that are highly expressed in the drought tolerant wild watermelon when exposed to drought and salinity stress is the drought-induced peptide (DRIP) (**Figure 2**). Yokota et al. (2002) exposed the watermelon cultivars and observed that DRIP-1 was induced in a similar manner in tolerance species response to either drought or salinity stress, thus suggesting it is highly involved in the tolerance mechanisms for the two stresses. A study on wild watermelon leaves under severe drought stress revealed new peptides (DRIP-1 to DRIP-6), with the two isoforms of DRIP-1 showing an abundant accumulation as compared to the other DRIPs (Kawasaki et al., 2000). This accumulation pattern of the peptides was not observed in the control plants and the DRIP-1 isoforms in the watermelon leaves was associated with the accumulation of drought tolerance amino acids like arginine, glutamate and citrulline which have shown to play a critical role in ornithine synthesis. An analysis of proteins on the wild watermelon leaves exposed to moisture deficit for three days showed higher accumulation of DRIP-1, which was 5 times higher compared to the domesticated watermelon (Yokota et al., 2002). Similarly Roy et al. (2009) observed several new DRIPs accumulation in tomato leaves exposed to moisture stress as compared to the control plants. The role of small peptides in salinity stress has also being studied and that peptide *AtPep13* (Nakaminami et al., 2018) and *BoPROPEPs* (Wang et al., 2022) was actively involved in the tolerance against salinity as they naturally increased when plants were exposed to salt stress. Zhou et al. (2022) observed that a small peptide (*PIP3*) played a significant role in salinity tolerance of Arabidopsis thaliana when mutant with PIP3 knocked out exhibited salt sensitivity as compared to the wild types.

Ornithine effects on aiding drought and salinity tolerance on plants have been studied and documented (Kalamaki et al., 2009b; Anwar et al., 2018; Hussein et al., 2019; Çavuşoğlu and Çavuşoğlu, 2021). Arginine and ornithine are also used for the synthesis of polyamines and some alkaloids in plants (Urbano-Gámez et al., 2020). The activity of small peptide protein and contribution to drought and salinity tolerance could be through polyamines and alkaloids. Strong antioxidant activity of these secondary metabolites suggests that they could be acting downstream of the peptide proteins as protectants against ROS.

Proteins are known to be involved in various signaling and acting as key transcriptional activators for living organisms when exposed to different environments (Fotovat et al., 2017; Priya et al., 2019). Study of proteomics in elucidating the response mechanisms of plants to environmental stress has had significant results, thus a continued use to evaluate other climatic resilient plants like wild watermelon is of continued importance. A proteomic analysis of wild watermelon after exposure to drought stress revealed several drought related proteins to be highly expressed (Akashi et al., 2011). A total of 23 stress induced and 6 stress repressed proteins were positively identified and of these were mostly the heat shock proteins (HSPs). The HSPs has extensively been proven to play an important part in living organisms when exposed to various biotic and abiotic stresses (Ruibal et al., 2013; Haq and Shakeel, 2020; Guo et al., 2020; Tian et al., 2021). Another proteomic study on wild watermelon by Si et al. (2009) revealed different expression of drought response related proteins when exposed to moisture stress with HSPs recording a high accumulation only four days when the crops were exposed to stress, while the NAC was highly expressed in the shoots as compared to the low expression observed in the roots when exposed to moisture stress. Several other proteins revealed varying expressions on either roots or shoots when exposed to moisture stress thus showing their active participation during drought response in wild watermelon. The role of response proteins in wild watermelon acclimatization to stress was further suggested by Li et al. (2018) who observed that the DEGs encoding late embryogenesis abundant (LEA) proteins, dehydrin, and heat shock proteins (HSPs) were highly expressed during drought stress.

These proteins that are highly associated with drought stress tolerance in wild watermelon and other crops have also shown to be active in tolerance to salinity stress (**Figure 2**). An analysis of the expressed proteins on wheat that was exposed to salinity revealed upregulation of several HSPs thus suggesting potential involvement in the salinity tolerance mechanism (Khateeb et al., 2020). To further ascertain the potential involvement of the HSPs in salinity tolerance mechanism, two HSP were isolated and overexpressed in rice and the results showed transgenic plants to have superior tolerance to both drought and salinity stress as compared to the wild type (Zou et al., 2021). A transcriptome analysis into the salt tolerance and sensitive willow genotypes recorded a total of 39 genes encoding for HSPs and all of these genes were upregulated under salinity stress and it was concluded that this small HSPs played in important role in response to salinity stress (Sui and Wang, 2020). However, some HSPs such as the watermelon ClHSP22.8 (He et al., 2021) could be key players in salinity tolerance and in water melons as negative regulators *via* ABA-dependent as well as independent and other signaling pathways. The involvement of these small HSPs in both drought and salinity stress are potential key players in the survival of plants under the two abiotic stresses. The body of knowledge accumulated so far has shown that HSPs play an important role wild watermelon tolerance to drought stress it can then be postulated that this HSPs could also contribute to the salinity stress when the plant is exposed to such as the two stresses could manifest through osmotic stress.

### Expression of GTPase

Small guanosine triphosphate (GTPases)-binding proteins are tightly regulated molecular switches in signal transduction. These are a group of independent superfamily within the class of regulatory GTP hydrolases and are proteins that are capable of controlling a number of important processes and they possess a common structurally conserved GTP-binding domain (Bourne et al., 1990; Nilsson et al., 2002; Liu and Huang, 2009). The super family comprises of four families (RAN, ARF, RAB, ROP) that are conserved across eukaryotes; where RAN has been implicated in nucleocytoplasmic transport, RNA synthesis, processing and export and cell cycle checkpoint control (Scheffzek et al., 1995; Rush et al., 1996; Khuperkar et al., 2015) and ROP being involved in actin dynamics regulation, which controls polar growth and root hair development (Luo et al., 2006) and in salt tolerance signal pathways in plants (Yang, 2002).

Even though the functions of GTPase in animals and yeast have been well characterized and documented, the same cannot be said about plants. With the few reports available, the RAN GTPases involvement in mitosis; cell division and proliferation has been suggested. Rose and Meier (2001) suggested that a domain unique to plant RANGAP (the RAN GTPase activating protein) is responsible for its target on the plant nuclear rim. Transcriptome studies have also revealed drought enrichment of GTPases in maize ovary tissues with respect to drought induced splicing (Kakumanu et al., 2012), regulating of stomatal opening (Wang et al., 2017), conferring maximum ABA sensitivity (Lee and Seo, 2019) in Arabidopsis, found to trigger innate immunity in rice (Kawano et al., 2010). When Ran GTPase was overexpressed in Arabidopsis and Rice, the transgenic plants showed distinct phenotypes like increased number of tillers, weak apical dominance, excess rosette leaves and abnormal root growth suggesting the involvement of RAN GTPase in cell proliferation (Wang et al., 2006; Xu and Cai, 2014);. A transgenic approach demonstrated that plants overexpressing RAN genes had enhanced cold (Xu et al., 2016) and osmotic stress tolerance (Akashi et al., 2016a) as transgenic plants maintained cell division under stress conditions. In another study, a Rop protein, OsRacB, which acts as an accessory regulatory component in salt stress responses was identified (Christensen, 2003) and it expression studied in rice. Transgenic rice treated with salinity stress grew much better than the control, suggesting that overexpression of OsRacB in rice partly improve salinity tolerance, as had been shown in tobacco (Luo et al., 2006).

In wild watermelon one of the distinct features during drought is in the root growth that can reach up-to 2-3 meters down the sandy soils in search of the little conserved moisture. This extensive root growth can be thought to be stimulated by the RAN GTPase during drought, as shown in the significant induction of the RAN GTPase during analysis of root proteome of wild watermelon in the early stages of the crop under drought stress (Yoshimura et al., 2008). When grown under PEG induced drought stress the roots of wild watermelon were significantly different from the domesticated watermelon in both length, hairy roots development and dry mass as the wild watermelon recorded 2-3 folds increases showing an extensive growth in wild watermelon root seedlings (Akashi et al., 2016a). Further, an overexpression of the CLRan on Arabidopsis resulted in increased root growth on both stressed and unstressed plants as compared to the wild types. A screening of the mRNA and protein abundance of the CLRan1 and CLRan2 displayed an incremental abundance of the RAN in the roots of the wild watermelon under drought stress as compared to the control. This thus suggests that the RAN GTPase is involved in the cell proliferation and this function is useful root development in wild watermelon thus playing a significant role when it comes to drought tolerance. These results thus further shows the involvement of small GTPases in both drought and salinity stress in plants. In response to salinity levels the grafted watermelon seedlings expressed several differentially expressed proteins and notably ATP synthase beta subunit was one of the protein highly expressed thus suggesting its role in the tolerance mechanisms against salinity stress (Yang et al., 2012).

### Expression of Metallothionein (MT) as a protectant

The Metallothioneins (MTs) are low molecular mass (7-10kDa) proteins, which have a higher percentage of cysteine amino acids in the sequence (Liu et al., 2014). These proteins have long been discovered and isolated from horse kidneys as cadmium-binding proteins (Margoshes and Vallee, 1957). Their expression has been documented in plant responses to oxidative stress, where they act as protective factors by protecting cells through scavenging of stress induced ROS (Ruttkay-Nedecky et al., 2013). The MTs are directly involved in the removal of ROS enhancing the protection against cellular injury (Nzengue et al., 2012) and also act as an antioxidant for ROS-induced cellular injury (Chiaverini and De Ley, 2010). The ROS accumulate to toxic levels during drought stress conditions (Malambane, 2018a).

When plants are exposed to abiotic stress a usual increase in expression of the MT gene is observed, thus it has been concluded that the gene is important in tolerance mechanisms as it is associated with cell growth and proliferation, detoxification and cellular repair while maintaining the cellular homeostasis through ROS scavenging (Kumar et al., 2012). Overexpression of the MT gene from date palm (Patankar et al., 2019) and *Suaeda salsa*, (Jin et al., 2017) conferred drought and salinity tolerance in yeast and Arabidopsis. The transgenic plants accumulated less Na+ and maintained a high K+/Na+ ratio, which could be attributed to the role of the transgene on transporters such as HKT (Patankar et al., 2019). Further Soda et al. (2016) observed that metallothionein strongly interacts with other proteins like cytoskeleton to improve abiotic (drought and salinity) in diverse plant species. This was further confirmed by Mekawy et al. (2018) and Kumar et al. (2012) in which overexpressing *Os*MT-3a and *Os*MT-1e-P showed an improved tolerance to the NaCl stress by scavenging ROS in rice, *E.coli* and tobacco.

An analysis of wild watermelon under moisture stress showed a number of up-regulated genes in the leaf, and among the up-regulated genes was the Type 2 MT designated as *Cl*MT2 (*Citrullus lanatus* metallothionein Type-2) (Akashi et al., 2004). The effects of *Cl*MT2 on protecting genomic DNA under moisture stress were investigated and the results showed that the DNA degradation was significantly suppressed by the *Cl*MT2 in a dose dependent manner (Akashi et al., 2004). These then suggest that the up-regulation of the *Cl*MT2 in the leaves aid the plants tolerance mechanisms through DNA protection by ROS scavenging when exposed to drought stress (**Figure 2**). This upregulation of the *Cl*MT in wild watermelon could suggest that the crop has a strong chance in withstanding salinity stress through the ROS scavenging thus protecting the internal cellular mechanisms for the plants.

## Conclusions and future perspectives

Drought stress is well documented to be a major factor in crop production and studies have shown that crops need a combination of factors to mitigate the effects of drought stress. Various studies on wild watermelon and other crops show that a combination of physiological, biochemical factors responses operating concert are responsible for drought tolerance. **Figure 2** illustrates the concerted morphological, physiological, and molecular traits that the watermelon has developed to aid the drought tolerance mechanisms. The responses are resilient root growth and timely stomatal closure, accumulation of citrulline and expression of genes associated with drought stress in watermelons and many other crops and model species. The mechanisms presented here can also be studied on other species with promising drought tolerance mechanisms and be compared to those of the wild watermelon. However, the mechanisms presented in this review might not be all that contributes to tolerance of the crop, thus more studies are needed for the species to be considered as a model reference crop for drought tolerance and related studies. With limited studies on the salinity tolerance on wild watermelon and relatives, this review then aimed to associate the mechanisms for drought tolerance to the potential the plant might have in tolerating salinity. We have observed through other studies that the mechanisms wild watermelon has shown as drought tolerance are also similar mechanisms other plant species confer in salinity stress. Thus it can be concluded that wild watermelon through the drought tolerance mechanism can also double up as salinity tolerance but this need to be conferred through a scientific research focusing on citrulline accumulation, its biosynthesis genes, APX and ROS scavenging responses to the two stresses.

Drought and salinity stress research involving watermelon at molecular level revealed established transcription factors for tolerance in others species. The transcription factors; AP2-ERBP, bZIP, MYB, NAC, WRKY and their downstream target genes, together regulons, have gained attention on their account of their role in drought and salinity tolerance pathways in plants. These are also presented in **Figure 2** as ABA-depended and independent responsive to drought and salinity stress. A recent trancriptome study by Zhu et al. (2022) revealed that in addition to these, the OFP and TCP family of transcription factors responded to salinity stream in watermelons. A growing body of evidence suggests that OFP and TCP transcription factors participate in drought and salinity stress pathways in other plants (Danisman, 2016; Wang et al., 2020). Further other small signaling proteins like the Aquaporins whose primary role has been suggested as to maintain the water homeostasis in living cells thus playing and important role in moisture related stresses by maintaining the cells osmotic potential (Vera-Estrella, 2004; Afzal et al., 2016). In their role against drought and salinity stresses, it has been suggested that the Aquaporins regulated changes in the root, stem and leaf hydraulic conductivity, plant water usage in response to stress (Vandeleur et al., 2009; Vandeleur et al., 2014; Sade and Moshelion, 2017). Similarly, an observation where an up regulation of *ClAQP* was observed under salinity and drought stress and also during fruit development of the cultivated watermelon and cucumis melo (Wang et al., 2016; Kuşvuran et al., 2021; Lopez-Zaplana et al., 2022) suggesting the involvement of the aquaporins in tolerance mechanisms of the citrullus species. Another important protectant that has been documented to play an important role in drought and salinity stress is the trehalose that is suggested to play an important role in regulation of stomatal aperture, regulation of plant water use, osmolyte protectants and acting as an energy source thus aiding the tolerance mechanisms of plants. This protectant mechanism has also been observed in the cultivated watermelon (Yuan et al., 2022a; Yuan et al., 2022b) where it aided the crop to survive against the drought and salinity stresses. Other important molecular mechanisms that has shown potential to play an important role in the tolerance mechanisms of citrullus species is the Thaumatin-like protein (TLP) that has been documented to be actively involved in responses abiotic stresses in watermelon (Ram et al., 2022). All these potential tolerance mechanisms as observed in cultivated watermelon could thus suggest a much more pronounced expression and role in the more tolerant wild watermelon. Thus, this calls for their attention on their role and that of their target genes, to establish new regulons common in drought and salinity stress in wild watermelon. Having identified the transcription factors transcriptome analyses, their validation for drought and salinity stress tolerance can be done in water melons through transgenesis, Virus-induced gene silencing (VIGS), RNA interference, clustered regularly interspaced short palindromic repeat (CRISPR) genome editing system. Application of these techniques will be enable high throughput drought and salinity tolerance gene discovery pathways enabled by availability of its genetic transformation protocols, genome sequence of 20 accessions representing three different *C. Lanatus* subspecies (subsp. *Vulgaris*, subsp. *Mucosospermus and* subsp. *Lanatus)* (Guo et al., 2012). The identified transcription factors can be used to develop drought and salinity tolerant transgenic plants across three subspecies and other crops. Furthermore, and owing to the availability of transcriptome and metabolomic analysis resources in water melons, elucidation of dynamic coordination of drought and salinity responsive transcription factors in interacting pathways and their specific integration in the cellular network will provide new opportunities for the engineering of plant tolerance to these stresses.

## Author contributions

GM conceptualized the research and wrote the manuscript; KM and LS conducted the literature review; UB conceptualized, supervised the research and reviewed the manuscript. All authors contributed to the article and approved the submitted version.

## References

[B1] Afzal Z. Howton T. C. Sun Y. Mukhtar M. S. (2016). The roles of aquaporins in plant stress responses. J. Dev. Biol. 4, 9. doi: 10.3390/jdb4010009 29615577 PMC5831814

[B2] Akashi K. Mifune Y. Morita K. Ishitsuka S. Tsujimoto H. Ishihara T. (2016b). Spatial accumulation pattern of citrulline and other nutrients in immature and mature watermelon fruits. J. Sci. Food Agric. 97, 479–487. doi: 10.1002/jsfa.7749 27060681

[B3] Akashi K. Miyake C. Yokota A. (2001). Citrulline, a novel compatible solute in drought tolerant wild watermelon leaves, is an efficient hydroxyl radical scavenger. FEBS Lett. 508, 438–442. doi: 10.1016/S0014-5793(01)03123-4 11728468

[B4] Akashi K. Nishimura N. Ishida Y. Yokota A. (2004). Potent hydroxyl radical-scavenging activity of drought-induced type-2 metallothionein in wild watermelon. Biochem. Biophys. Res. Commun. 323, 72–78. doi: 10.1016/j.bbrc.2004.08.056 15351703

[B5] Akashi K. Yoshida K. Kuwano M. Kajikawa M. Yoshimura K. Hoshiyasu S.. (2011). Dynamic changes in the leaf proteome of a C3 xerophyte, citrullus lanatus (wild watermelon), in response to water deficit. Planta 233, 947–960. doi: 10.1007/s00425-010-1341-4 21259065

[B6] Akashi K. Yoshimura K. Kajikawa M. Hanada K. Kosaka R. Kato A.. (2016a). Potential involvement of drought-induced ran GTPase CLRan1 in root growth enhancement in a xerophyte wild watermelon. Biosci Biotechnol. Biochem. 80, 1907–1916. doi: 10.1080/09168451.2016.1191328 27310473

[B7] Akashi K. Yoshimura K. Nanasato Y. Takahara K. Munekage Y. Yokota A. (2008). Wild plant resources for studying molecular mechanisms of drought/strong light stress tolerance. Plant Biotechnol. 25, 257–263. doi: 10.5511/plantbiotechnology.25.257

[B8] An P. Inanaga S. Li X. Shimizu H. Tanimoto E. (2003). Root characteristics in salt tolerance. Root Res. 12, 125–132. doi: 10.3117/rootres.12.125

[B9] Anwar S. Ali M. A. Inselsbacher E. (2021). Phenotypic analysis of arabidopsis thaliana arginine-deficient mutants. Pure Appl. Biol. (PAB) 11, 302–314. doi: 10.19045/bspab.2022.110032

[B10] Anwar A. She M. Wang K. Riaz B. Ye X. (2018). Biological roles of ornithine aminotransferase (OAT) in plant stress tolerance: Present progress and future perspectives. Int. J. Mol. Sci. 19, 3681. doi: 10.3390/ijms19113681 30469329 PMC6274847

[B11] Arif M. R. Islam M. T. Robin A. H. K. (2019). Salinity stress alters root morphology and root hair traits in brassica napus. Plants (Basel) 8, 192. doi: 10.3390/plants8070192 31252515 PMC6681291

[B12] Arif H. K. B. Matthew C. Uddin M. J. Bayazid K. N. (2016). Salinity-induced reduction in root surface area and changes in major root and shoot traits at the phytomer level in wheat. J. Exp. Bot. 67, 3719–3729. doi: 10.1093/jxb/erw064 26951370

[B13] Asada K. (1992). Ascorbate peroxidase - a hydrogen peroxide-scavenging enzyme in plants. Physiologia Plantarum 85, 235–241. doi: 10.1111/j.1399-3054.1992.tb04728.x

[B14] Ashraf M. Y. Akhtar K. Sarwar G. Ashraf M. (2005). “Role of the rooting system in salt tolerance potential of different guar accessions,” in Agronomy for sustainable development, vol. 25. (Springer Verlag/EDP Sciences/INRA), 243–249.

[B15] Baker N. R. (2008). Chlorophyll fluorescence: a probe of photosynthesis *in vivo* . Annu. Rev. Plant Biol. 59, 89–113. doi: 10.1146/annurev.arplant.59.032607.092759 18444897

[B16] Badawi G. H. Kawano N. Yamauchi Y. Shimada E. Sasaki R. Kubo A. (2004). Over-expression of ascorbate peroxidase in tobacco chloroplasts enhances the tolerance to salt stress and water deficit. Physiol Plant. 121, 231–238. doi: 10.1111/j.0031-9317.2004.00308.x 15153190

[B17] Baker N. R. Rosenquist E. (2004). Applications of chlorophyll flourescemce can improve crop production strategies: An examination of future possibilities. J. Exp. Bot. 55, 1607–1621. doi: 10.1093/jxb/erh196 15258166

[B18] Bao Y. Aggarwal P. Robbins N. E. II Sturrock C. J. Thompson M. C. Tan H. Q.. (2014). Plant roots use a patterning mechanism to position lateral root branches toward available water. PNAS 111, 9319–9324. doi: 10.1073/pnas.1400966111 24927545 PMC4078807

[B19] Baxter C. J. Redestig H. Schauer N. Repsilber D. Patil K. R. Nielsen J.. (2007). The metabolic response of heterophic arabidopsis cells to oxidative stress. Plant Physiol. 143, 312–325. doi: 10.1104/pp.106.090431 17122072 PMC1761969

[B20] Bourne H. R. Sanders D. A. McCormick F. (1990). The GTPase superfamily: a conserved switch for diverse cell functions. Nature 348, 125–132. doi: 10.1038/348125a0 2122258

[B21] Brown C. Ghile Y. Laverty M. Li K. (2012). Decision scaling: Linking bottom-up vulnerability analysis with climate projections in the water sector. Water Resour. Res. 48, W09537. doi: 10.1029/2011WR011212

[B22] Cao D. Lutz A. Hill C. B. Callahan D. L. Roessner U. (2017). A quantitative profiling method of phytohormones and other metabolites applied to barley roots subjected to salinity stress. Front. Plant Sci. 7, 2070. doi: 10.3389/fpls.2016.02070 28119732 PMC5222860

[B23] Caverzan A. Passaia G. Rosa S. B. Ribeiro C. W. Lazzarotto F. Margis-pinheiro M. (2012). Plant response to stresses: Role of ascorbate peroxidase in the antioxidant protection. Genet. Mol. Biol. 35, 1011–1019. doi: 10.1590/S1415-47572012000600016 23412747 PMC3571416

[B24] Çavuşoğlu K. Çavuşoğlu D. (2021). Role of l-ornithine in mitigation of salt stress in allium cepa l. Bangladesh J. Bot. 50, 1165–1171. doi: 10.3329/bjb.v50i4.57085

[B25] Chang J. Cheong B. E. Natera S. Roessner U. (2019). Morphological and metabolic responses to salt stress of rice (Oryza sativa l.) cultivars which differ in salinity tolerance. Plant Physiol. Biochem. 144, 427–435. doi: 10.1016/j.plaphy.2019.10.017 31639558

[B26] Chen Y. L. Fan K. T. Hung S. C. Chen Y. T. (2019). The role of peptides cleaved from proteins precursors in eliciting plants stress reactions. New Phytol. 6, 2267–2282. 10.1111/nph.16241 31595506

[B27] Chen Y. Li C. Zhang B. Yi J. Yang Y. Kong C.. (2019). The role of the late embryogenesis-abundant (LEA) protein family in development and the abiotic stress response: A comprehensive expression analysis of potato (Solanum tuberosum). Genes (Basel) 10, 148. doi: 10.3390/genes10020148 30781418 PMC6410179

[B28] Chiaverini N. De Ley M. (2010). Protective effect of metallothionein on oxidative stress-induced DNA damage. Free Radicals Res. 44, 605–613. doi: 10.3109/10715761003692511 20380594

[B29] Choudhury S. Mansi Muthusamy S. K. Padaria J. C. Dalal M. (2021). Genome-wide identification of ran GTPase family genes from wheat (T. aestivum) and their expression profile during developmental stages and abiotic stress conditions. Funct. Integr. Genomics 21, 239–250. doi: 10.1007/s10142-021-00773-0 33609188

[B30] Christensen T. M. Vejlupkova Z. Sharma K. Y. Arthur K. M. Spatafora W. J. Albright A. C.. (2003). Conserved subgroups and developmental regulation in the monocot rop gene family. Plant Physiol. 133, 1791–1808. doi: 10.1104/pp.103.029900 14605221 PMC300733

[B31] Comas L. H. Becker S. R. Cruz V. M. V. Byrne P. F. Dierig D. A. (2013). Root traits contributing to plant productivity under drought. Front. Plant Sci. 4, 442. doi: 10.3389/fpls.2013.00442 24204374 PMC3817922

[B32] Condon A. G. Hall A. E. (1997). 3-adaptation to diverse environments; variations in water-use efficiency within crop species. Environ. Sci. (Academic Press), 79–116. doi: 10.1016/B978-012378260-1/50004-X

[B33] Cornic G. Massacci A. (1996). “Leaf photosynthesis under drought stress,” in Photosynthesis and the environment. Ed. Baker N. R. (The Netherlands: Kluwer Academic Publishers).

[B34] Czyzewicz N. Shi C. L. Vu L. D. Van De Cotte B. Hodgman C. Butenko M. A. (2015). Modulation of arabidopsis and monocot root architecture by CLAVATA3/EMBRYO SURROUNDING REGION 26 peptide. J. Exp. Bot. 66, 5229–5243. doi: 10.1093/jxb/erv360 26188203 PMC4526925

[B35] Danisman S. (2016). TCP Transcription factors at the interface between environmental challenges and the plant’s growth responses. Front. Plant Sci. 7, 1930. doi: 10.3389/fpls.2016.01930 28066483 PMC5174091

[B36] D’Arcy-Lameta A. Ferrari-Iliou R. Contour-Ansel D. Pham-Thi A. T. Zuily-Fodil Y. (2006). Isolation and characterization of four ascorbate peroxidase cDNAs responsive to water deficit in cowpea leaves. Ann. Bot. 97, 133–140. doi: 10.1093/aob/mcj010 16311273 PMC2000772

[B37] Dasgan H. Y. Kusvuran S. Abak K. Leport L. Larher F. Bouchereau A. (2009). The relationship between citrulline accumulation and salt tolerance during the vegetative growth of melon (Cucumis melo l.). Plant Soil Environ. 55, 51–57. doi: 10.17221/316-PSE

[B38] Daszkowska-Golec A. Szarejko. I. (2013). Open or close the gate – stomata action under the control of phytohormones in drought stress conditions. Front. Plant Sci. 4, 138. doi: 10.3389/fpls.2013.00138 23717320 PMC3652521

[B39] Demmig-Adams B. Adams W. W. (2006). Photoprotection in an ecological context: the remarkable complexity of thermal energy dissipation. New Phytol 172, 11–21. doi: 10.1111/j.1469-8137.2006.01835.x 16945085

[B40] Desikan R. H.-Mackerness S. A. Hancock J. T. Steven J. N. (2001). Regulation of the arabidopsis transcriptome by oxidative stress. Plant Physiol. 127, 159–172. doi: 10.1104/pp.127.1.159 11553744 PMC117972

[B41] Diaz-Vivancos P. Barba-Espín G. Hernández J. A. (2013). Elucidating hormonal/ROS networks during seed germination: insights and perspectives. Plant Cell Rep. 32, 1491–502. doi: 10.1007/s00299-013-1473-7 23812175

[B42] Dietz K. J. Tavakoli N. Kluge C. Mimura T. Sharma S. S. Harris G. C.. (2001). Significance of the V-type ATPase for the adaptation to stressful growth conditions and its regulation on the molecular and biochemical level. J. Exp. Bot. 52, 1969–1980. doi: 10.1093/jexbot/52.363.1969 11559732

[B43] Du M. Zhai Q. Deng L. Li S. Li H. Yan L.. (2014). Closely related NAC transcription factors of tomato differentially regulate stomatal closure and reopening during pathogen attack. Plant Cell 26, 3167–3184. doi: 10.1105/tpc.114.128272 25005917 PMC4145139

[B44] Dourado P. R. M. de Souza E. R. Santos M. A. D. Lins C. M. T. Monteiro D. R. Paulino M. K.S. S. (2022). Stomatal Regulation and Osmotic Adjustment in Sorghum in Response to Salinity. Agriculture 12, 658. doi: 10.3390/agriculture12050658

[B45] Escalona J. M. Flexas J. Medrano H. (2000). Stomatal and non-stomatal limitations to photosynthesis under water stress in field-grown grapevines. Aust. J. Plant Physiol. 27, 87–87. doi: 10.1071/PP99019_CO

[B46] Farooq M. Wahid A. Kobayashi N. Fujita D. Basra S. M. A. (2008). Plant drought stress: effects, mechanisms and management. Agron. Sustain. Dev. 29, 185–212. doi: 10.1051/agro:2008021

[B47] Farquhar G. D. Sharkey T. D. (1982). Stomatal conductance and photosynthesis. Annu. Rev. Plants Biol. 61, 561–591. doi: 10.1146/annurev.pp.33.060182.001533

[B48] Feirs M. Golemiec E. Xu J. van der Geest L. Heidstra R. Stiekema W.. (2005). The 14-amino acids CLV3, CLE19, and CLE40 peptides trigger consumption of the root meristems in arabidopsis through a CLAVATA-dependent pathway. Plant Cell 17, 2542–2553. doi: 10.1105/tpc.105.034009 16055633 PMC1197433

[B49] Feng X. Jia L. Cai Y. Guan H. Zheng D. Zhang W.. (2022). ABA inducible DEEPER ROOTING 1 improves adaptation of maize to water deficiency. Plant Biotechnol. J. 20, 2077–2088. doi: 10.1111/pbi.13889 35796628 PMC9616520

[B50] Fini A. Guidi L. Ferrini F. Brunetti C. di Ferdinando M. Biricolti S.. (2012). Drought stress has contrasting effects on antioxidant enzymes activity and phenylpropanoid biosynthesis in fraxinus ornus leaves: An excess light stress affair? J. Plant Physiol. 169, 929–939. doi: 10.1016/j.jplph.2012.02.014 22537713

[B51] Fotovat R. Alikhani M. Valizadeh M. Mirzaei M. Salekdeh G. H. (2017). A proteomics approach to discover drought tolerance proteins in wheat pollen grain at meiosis stage. Protein Peptides Lett. 24, 26–36. doi: 10.2174/0929866523666161130143446 27908260

[B52] Garg R. Shankar R. Thakkar B. Kudapa H. Krishnamurthy L. Mantri N.. (2016). Transcriptome analyses reveal genotype- and developmental stage-specific molecular responses to drought and salinity stresses in chickpea. Sci. Rep. 6, 19228. doi: 10.1038/srep19228 26759178 PMC4725360

[B53] Gavassi M. A. Silva G. S. Santiago G. M. Thompson A. J. Macleod K. Oliveira P. M. R.. (2021). NCED expression is related to increased ABA biosynthesis and stomatal closure under aluminum stress. Environ. Exp. Bot. 185, 104404. doi: 10.1016/j.envexpbot.2021.104404

[B54] Ghorecha V. Zheng Y. Liu L. Sunkar R. Krishnayya N. S. R. (2017). MicroRNA dynamics in a wild and cultivated species of convolvulaceae exposed to drought stress. Physiol. Mol. Biol. Plants 23, 291–300. doi: 10.1007/s12298-017-0426-y 28461718 PMC5391358

[B55] Gibson A. C. (1996). Origins of desert structural adaptations. In: Structure-function relations of warm desert plants. Adaptations of desert organisms. Berlin, Heidelberg: Springer. doi: 10.1007/978-3-642-60979-4_7

[B56] Gifford M. L. Banta J. A. Katari M. S. Hulsmans J. Chen L. Ristova D.. (2013). Plasticity regulators modulate specific root traits in discrete nitrogen environments. PloS Genet. 9, e1003760. doi: 10.1371/journal.pgen.1003760 24039603 PMC3764102

[B57] Gorim L. Y. Vandenberg A. (2017). Evaluation of wild lentil species as genetic resources to improve drought tolerance in cultivated lentil. Front. Plant Sci. 8, 1129. doi: 10.3389/fpls.2017.01129 28706524 PMC5489631

[B58] Guan Q. Wang Z. Wang X. Takano T. Liu S. (2015). A peroxisomal APX from Puccinellia tenuiflora improves the abiotic stress tolerance of transgenic Arabidopsis thaliana through decreasing of H2O2 accumulation. J Plant Physiol. 175, 183–191. doi: 10.1016/j.jplph.2014.10.020 25644292

[B59] Guidi L. Lo Piccolo E. Landi M. (2019). Chlorophyll fluorescence, photoinhibition and abiotic stress: Does it make any difference the fact to be a C3 or C4 species? Front. Plant Sci. 10, 174. doi: 10.3389/fpls.2019.00174 30838014 PMC6382737

[B60] Guochao X. Congming L. Ruofang Z. Jiming J. (2017). Overexpression of StNF-YB3.1 reduces photosynthetic capacity and tuber production, and promotes ABA-mediated stomatal closure in potato (Solanum tuberosum l.). Plant Sci. 261, 50–59. doi: 10.1016/j.plantsci.2017.04.015 28554693

[B61] Guo J. Sun B. He H. Zhang Y. Tian H. Wang B. (2021). Current understanding of bHLH transcription factors in plant abiotic stress tolerance. Int. J. Mol. Sci. 22, 4921. doi: 10.3390/ijms22094921 34066424 PMC8125693

[B62] Guo L. M. Li J. He J. Liu H. Zhang H. (2020). A class I cytosolic HSP20 of rice enhances heat and salt tolerance in different organisms. Sci Rep 10, 1383. doi: 10.1038/s41598-020-58395-8 31992813 PMC6987133

[B63] Guo S. Zhang J. Sun H. Salse J. Lucas W.J. Zhang H. (2012). The draft genome of watermelon (Citrullus lanatus) and resequencing of 20 diverse accessions. Nat. Genet. 45, 51–58. doi: 10.1038/ng.2470 23179023

[B64] Guseman J. M. Webb K. Srinivasan C. Dardick C. (2017). DRO1 influences root system architecture in arabidopsis and prunus species. Plant J. 89, 1093–1105. doi: 10.1111/tpj.13470 28029738

[B65] Haq N. U. Shakeel S. N. (2020). HSPs under Abiotic Stresses. In Fahad S. Saud S. Chen Y. Wu C. Wang D. (Eds.), Abiotic Stress in Plants. London: IntechOpen. doi: 10.5772/intechopen.93787

[B66] He Y. Yao Y. Li L. Li Y. Gao J. Fan M. (2021). A heat-shock 20 protein isolated from watermelon (ClHSP22.8) negatively regulates the response of arabidopsis to salt stress *via* multiple signaling pathways. Peer J. 9, e10524. doi: 10.7717/peerj.10524 33717662 PMC7931717

[B67] Hernández J. A. Ferrer M. A. Jiménez A. Barceló A. R. Sevilla F. (2001). Antioxidant systems and O2.-/H2O2 production in the apoplast of pea leaves. Its relation with salt-induced necrotic lesions in minor veins. Plant Physiol. 127, 817–831. doi: 10.1104/pp.010188 11706165 PMC129254

[B68] Hniličková H. Hnilička F. Martinkova J. Kraus K. (2017). Effects of salt stress on water status, photosynthesis and chlorophyll fluorescence of rocket. Plant Soil Environ. 63, 362–367. doi: 10.17221/398/2017-PSE

[B69] Horton P. Ruban A. V. (2005). Molecular design of the photosystem II light-harvesting antenna: photosynthesis and photoprotection. J. Exp. Bot. 56, 365–373. doi: 10.1093/jxb/eri023 15557295

[B70] Hoshiyasu S. Kohzuma K. Yoshida K. Fujiwara M. Fukao Y. Yokota A.. (2013). Potential involvement of n-terminal acetylation in the quantitative regulation of the " subunit of chloroplast ATP synthase under drought stress. Biosci Biotechnol. Biochem. 77, 998–1007. doi: 10.1271/bbb.120945 23649264

[B71] Hossain M. D. M. Hon-Ming L. Zhang J. (2015). Responses in gas exchange and water status between drought-tolerant and -susceptible soybean genotypes with ABA application. Crop J. 3, 500–506. doi: 10.1016/j.cj.2015.09.001

[B72] Hou W. Sun A. H. Chen H. L. Yang F. S. Pan J. L. Guan M. Y. (2016). Effects of chilling and high temperatures on photosynthesis and chlorophyll fluorescence in leaves of watermelon seedlings. Biol. Plantarum 1, 148–154. doi: 10.1007/s10535-015-0575-1

[B73] Hussain M. I. Dionyssia-Angeliki L. Farooq M. Nikoloudakis N. Khalid N. (2015). Salt and drought stresses in safflower: a review. Agron. Sustain. Dev. 36, 4. doi: 10.1007/s13593-015-0344-8

[B74] Hussein H. B. A. Mekki B. B. E.Abd El-Sadek M. Ebd El Lateef E. (2019). Effect of l-ornithine application on improving drought tolerance in sugar beet plants. Heliyon 5, e02631. doi: 10.1016/j.heliyon.2019.e02631 31667428 PMC6812460

[B75] Iseki K. Takahashi Y. Muto C. Naito K. Tomooka N. (2018). Diversity of drought tolerance in the genus vigna. Front. Plant Sci. 9, 729. doi: 10.3389/fpls.2018.00729 29963062 PMC6014140

[B76] Jaleel C. A. Manivannan P. Wahid A. Farooq M. Somasundaram R. Panneerselvam R. (2009). Drought stress in plants: a review on morphological characteristics and pigments composition. Int. J. Agric. Biol. 11, 100–105.

[B77] Janagoudar B. S. (2007). Salinity induced changes in stomatal response, bio-physical parameters, solute accumulation and growth in cotton (Gossypium spp.) The World Cotton Research Conference-4 September 10-14, 2007. Texas, USA: Lubbock Memorial Civic Center.

[B78] Jia M. Li D. Colombo R. Wang Y. Wang X. Cheng T.. (2019). Quantifying chlorophyll fluorescence parameters from hyperspectral reflectance at the leaf scale under various nitrogen treatment regimes in winter wheat. Remote Sens. 11, 2838. doi: 10.3390/rs11232838

[B79] Jiang J. Ma S. Ye N. Jiang M. Cao J. Zhang J. (2017). WRKY transcription factors in plant responses to stresses. J. Integrated Plant Biol. 59, 86–101. doi: 10.1111/jipb.12513 27995748

[B80] Jin S. Xu C. Li G. Sun D. Li Y. Wang X.. (2017). Functional characterization of a type 2 metallothionein gene, SsMT2, from alkaline tolerant suaeda salsa. Sci. Rep. 7, 17914. doi: 10.1038/s41598-017-18263-4 29263347 PMC5738349

[B81] Jin L. Yarra R. Zhou L. Cao H. (2022). The auxin response factor (ARF) gene family in oil palm (Elaeis guineensis jacq.): Genome-wide identification and their expression profiling under abiotic stresses. Protoplasma 259, 47–60. doi: 10.1007/s00709-021-01639-9 33792785

[B82] Joshi V. Fernie A. R. (2017). Citrulline metabolism in plants. Amino Acids 49, 1543–1559. doi: 10.1007/s00726-017-2468-4 28741223

[B83] Kajikawa M. Morikawa K. Abe Y. Yokota A. Akashi K. (2010). Establishment of a transgenic hairy root system in wild and domesticated watermelon (Citrullus lanatus) for studying root vigor under drought. Plant Cell Rep. 29, 771–778. doi: 10.1007/s00299-010-0863-3 20445980

[B84] Kakumanu A. Ambavaram M. M. R. Klumas C. Krishnan A. Batlang U. Myers E.. (2012). Effects of drought on gene expression in maize reproductive and leaf meristem tissue revealed by RNA-seq. Plant Physiol. 160, 846–867. doi: 10.1104/pp.112.200444 22837360 PMC3461560

[B85] Kalaji H. M. Govindjee B. K. Kościelniak J. Żuk-Golaszewska K. (2011). Effects of salt stress on photosystem II efficiency and CO2 assimilation of two Syrian barley landraces. Environ. Exp. Bot. 73, 64–72. doi: 10.1016/j.envexpbot.2010.10.009

[B86] Kalamaki M. S. Alexandrou D. Lazari D. Merkouropoulos G. Fotopoulos V. Pateraki I.. (2009b). Over-expression of a tomato n-acetyl-L-glutamate synthase gene (SlNAGS1) in arabidopsis thaliana results in high ornithine levels and increased tolerance in salt and drought stresses. J. Exp. Bot. 60, 1859–1871. doi: 10.1093/jxb/erp072 19357433 PMC2671631

[B87] Kalamaki M. S. Merkouropoulos G. Kanellis A. K. (2009a). Can ornithine accumulation modulate abiotic stress tolerance in arabidopsis? Plant Signal Behav. 4, 1099–1111. doi: 10.4161/psb.4.11.9873 19901538 PMC2819526

[B88] Kausar R. Hossain Z. Makino T. Komatsu S. (2012). Characterization of ascorbate peroxidase in soybean under flooding and drought stresses. Mol. Biol. Rep. 39, 10573–10579. doi: 10.1007/s11033-012-1945-9 23053956

[B89] Kawano Y. Chen L. Shimamoto K. (2010). The function of rac small GTPase and associated proteins in rice innate immunity. Rice 3, 112–121. doi: 10.1007/s12284-010-9049-4

[B90] Kawasaki S. Miyake C. Kohchi T. Fujii S. Uchida M. Yokota A. (2000). Responses of wild watermelon to drought stress: accumulation of an ArgE homologue and citrulline in leaves during water deficits. Plant Cell Physiol. 41, 864–873. doi: 10.1093/pcp/pcd005 10965943

[B91] Karahara I. Horie T. (2021). Functions and structure of roots and their contributions to salinity tolerance in plants. Breed Sci. 71 (1), 89–108. doi: 10.1270/jsbbs.20123 33762879 PMC7973495

[B92] Khan N. Bano A. Rahman M. A. Guo J. Kang Z. Babar M. A. (2019). Comparative physiological and metabolic analysis reveals a complex mechanism involved in drought tolerance in chickpea (Cicer arietinum l.) induced by PGPR and PGRs. Sci. Rep. 9, 2097. doi: 10.1038/s41598-019-38702-8 30765803 PMC6376124

[B93] Khateeb W. Muhaidat R. Alahmed S. Al Zoubi M. S. Al-Batayneh K. M. El-Oqlah A.. (2020). Heat shock proteins gene expression and physiological responses in durum wheat (Triticum durum) under salt stress. Physiol Mol Biol Plants. 26, 1599–1608. doi: 10.1007/s12298-020-00850-x 32801489 PMC7415065

[B94] Khuperkar D. Helen M. Magre I. Joseph J. (2015). Inter-cellular transport of ran GTPase. PloS One 10, e0125506. doi: 10.1371/journal.pone.0125506 25894517 PMC4403925

[B95] Kim J. S. Jeon B. W. Kim J. (2021). Signaling Peptides Regulating Abiotic Stress Responses in Plants. Front. Plant Sci. 12, 704490. doi: 10.3389/fpls.2021.704490 34349774 PMC8326967

[B96] Kohzuma K. Akashi K. Munekage Y. N. Sanda S. Hisabori T. Yokota A. (2008). “Preferential decay of the CF1-e subunit induces thylakoid uncoupling in wild watermelon under drought stress,” in Photosynthesis. energy from the sun: 14th international congress on photosynthesis. Eds. Allen J. F. Gantt E. Golbeck J. H. Osmond B. (Springer), 617–621.

[B97] Kohzuma K. Cruz J. A. Akashi K. Hoshiyasu S. Munekage Y. N. Yokota A.. (2009). The long-term responses of the photosynthetic proton circuit to drought. Plant Cell Environ. 32, 209–219. doi: 10.1111/j.1365-3040.2008.01912.x 19021886

[B98] Komura M. Yamagishi A. Shibata Y. Iwasaki I. Itoh S. (2010). Mechanism of strong quenching of photosystem II chlorophyll fluorescence under drought stress in a lichen, physciella melanchla, studied by subpicosecond fluorescence spectroscopy, biochimica et biophysica acta (BBA). Bioenergetics 1797, 331–338. doi: 10.1016/j.bbabio.2009.11.007 19962955

[B99] Kulkarni M. Soolanayakanahally R. Ogawa S. Uga Y. Selvaraj M. G. Kagale S. (2017). Drought response in wheat: Key genes and regulatory mechanisms controlling root system architecture and transpiration efficiency. Frontier Chem. 5, 106. doi: 10.3389/fchem.2017.00106 PMC5723305 29259968

[B100] Kumar G. Kushwaha H. R. Panjabi-Sabharwal V. Kumari S. Joshi R. Mittal S.. (2012). Clustered metallothionein genes are co-regulated in rice and ectopic expression of OsMT1e-p conferes multiple abiotic stress tolerance in tobacco *via* ROS scavenging. BMC Plant Biol. 12, 107. doi: 10.1186/1471-2229-12-107 22780875 PMC3491035

[B101] Kuşvuran Ş Kaya E. Ellialtıoğlu Ş. (2021). Role of Grafting in Tolerance to Salt Stress in Melon (Cucumis melo L.) Plants: Ion regulation and antioxidant defense systems. Biotechnol. Stud. 30, 22–32. doi: 10.38042/biotechstudies.932376

[B102] Kuşvuran S. Dasgan H. Y. Abak K. (2013). Citrulline is an important biochemical indicator in tolerance to saline and drought stresses in melon. Sci. World J. 2013, 253414. doi: 10.1155/2013/253414 PMC3864137 24363615

[B103] Laffray D. Louguet P. (1990). Stomatal response and drought resistance. Bull. la Société botanique france. Actualités Botaniques 137, 47–60. doi: 10.1080/01811789.1990.10826986

[B104] Lay F. T. Anderson M. A. (2005). Defensins- components of the innate immune systems in plants. Curr. Protein Peptides Sci. 6, 85–101. doi: 10.2174/1389203053027575 15638771

[B105] Lazzarotto F. Teixeira F. K. Rosa S. B. Dunand C. Fernandes C. L. de Vasconcelos Fontenele A.. (2011). Ascorbate peroxidase-related (APx-r) is a new heme-containing protein functionally associated with ascorbate peroxidase but evolutionarily divergent. New Phytol. 191, 234–250. doi: 10.1111/j.1469-8137.2011.03659.x 21352234

[B106] Lee S. C. Lee W. K. Ali A. Kumar M. Yang T. J. Song K. (2017). Genome-wide identification of the dehydrin genes in the cucurbitaceae species. Plant Breed. Biotechnol. 5, 282–292. doi: 10.9787/PBB.2017.5.4.282

[B107] Lee H. G. Seo P. J. (2019). MYB96 recruits the HDA15 protein to suppress negative regulators of ABA signaling in arabidopsis. Nat. Commun. 10, 1713. doi: 10.1038/s41467-019-09417-1 30979883 PMC6461653

[B108] Li J. Han Y. Liu L. Chen Y. Du Y. Zhang J.. (2015). Qrt9, a quantitative trait locus controlling root thickness and root length in upland rice. J. Exp. Bot. 66, 2723–2732. doi: 10.1093/jxb/erv076 25769309

[B109] Li Q. Li P. Sun L. Wang Y. Ji K. Sun Y.. (2012). Expression analysis of β-glucosidase genes that regulate abscisic acid homeostasis during watermelon (Citrullus lanatus) development and under stress conditions. J. Plant Physiol. 169, 78–85. doi: 10.1016/j.jplph.2011.08.005 21940067

[B110] Li H. B. Ma Q. H. Li H. G. Zhang F. S. Rengel Z. Shen J. B. (2014). Root morphological responses to localized nutrient supply differ among crop species with contrasting root traits. Plant Soil 376, 151–163. doi: 10.1007/s11104-013-1965-9

[B111] Li J. Li Y. Yin Z. Jiang J. Zhang M. Guo X.. (2017). OsASR5 enhances drought tolerance through a stomatal closure pathway associated with ABA and H2 O2 signalling in rice. Plant Biotechnol J. 15, 183–196. doi: 10.1111/pbi.12601 27420922 PMC5258865

[B112] Li H. Mo Y. Cui Q. Yang X. Guo Y. Wei C.. (2018). Transcriptomic and physiological analyses reveal drought adaptation strategies in drought-tolerant and -susceptible watermelon genotypes. Plant Sci. 18, 30747–30747. doi: 10.1016/j.plantsci.2018.10.016 30471727

[B113] Lin K. H. Pu S. F. (2010). Tissue and genotype specific ascorbate peroxidase expression in sweet potato in response to salt stress. Biol Plantarum 54, 664–670.

[B114] Liu K. Huang Y. (2009). RanGTPase: A key regulator of nucleo-cytoplasmic trafficking. Mol. Cell Pharamacol. 1, 148–156. doi: 10.4255/mcpharmacol.09.19 PMC2839366 20300488

[B115] Liu S. Liu S. Wang M. Meng C. Wang M. Xia G. (2014). A wheat SIMILAR TO RCD-ONE gene enhances seedling growth and abiotic stress resistance by modulating redox homeostasis and maintaining genomic integrity. Plant Cell 26, 164–180. doi: 10.1105/tpc.113.118687 24443520 PMC3963566

[B116] Liu H. Shen J. Yuan C. Lu D. Acharya B. R. Wang M.. (2021). The cyclophilin ROC3 regulates ABA-induced stomatal closure and the drought stress response of arabidopsis thaliana. Front. Plant Sci. 107, 713–736. doi: 10.3389/fpls.2021.668792 PMC8186832 34113366

[B117] Liu Y. Wu H. Kou L. Liu X. Zhang J. Guo Y.. (2014). Two metallothionein genes in oxya chinensis: Molecular characteristics, expression patterns and roles in heavy metal stress. PloS One 9, e112759. doi: 10.1371/journal.pone.0112759 25391131 PMC4229212

[B118] Li H. Xu G. Yang C. Yang L. Liang Z. (2019). Genome-wide identification and expression analysis of HKT transcription factor under salt stress in nine plant species. Ecotoxicol. Environ. Saf. 171, 435–442. doi: 10.1016/j.ecoenv.2019.01.008 30639869

[B119] Lopez-Zaplana A. Martinez-Garcia N. Carvajal M. Bárzana G. (2022). Relationships between aquaporins gene expression and nutrient concentrations in melon plants (Cucumis melo l.) during typical abiotic stresses. Environ. Exp. Bot. 195, 104759. doi: 10.1016/j.envexpbot.2021.104759

[B120] Luo M. Gu S. H. Zhao S. H. Zhang F. Wu N. H. (2006). Rice GTPase OsRacB: potential accessory factor in plant salt-stress signaling. Acta Biochim. Biophys. Sin. 38, 393–402. doi: 10.1111/j.1745-7270.2006.00172.x 16761097

[B121] Lu C. Zhang J. (2000). Role of light in theresponse of PSII photochemistry to salt stress inthe cyanobacterium spirulina platensis. J. Exp. Bot. 51, 911–917. doi: 10.1093/jxb/51.346.911 10948217

[B122] Lu C. Qiu N. Wang B. Zhang J. (2003). Salinity treatment shows no effects on photosystem II photochemistry, but increases the resistance of photosystem II to heat stress in halophyte Suaeda salsa. J. Exp. Bot. 54, 851–860. doi: 10.1093/jxb/erg080 12554728

[B123] Lynch J. P. (2007). Roots of the second green revolution. Aust. J. Bot. 55, 493–512. doi: 10.1071/BT06118

[B124] Malambane G. (2018a). Research on drought physiology, molecular responses, and development of biotechnology tools for the drought-tolerant wild watermelon (Citrullus lanatus acc.101117-1) (Tottori University, Japan: PhD Thesis).

[B125] Malambane G. Batlang U. Ramolekwa K. Tsujimoto H. Akashi K. (2021). Growth chamber and field evaluation of physiological factors of two watermelon genotypes. Plant Stress 2, 100017. doi: 10.1016/j.stress.2021.100017

[B126] Malambane G. Tsujimoto H. Akashi K. (2018b). The cDNA structures and expression profile of the ascorbate peroxidase family during drought stress in wild watermelon. J. Agric. Sci. 8, 56–72. doi: 10.5539/jas.v10n8p56

[B127] Malamy J. C. (2005). Intrinsic and environmental response pathways that regulate root system architecture. Plant Cell Environ. 28, 67–77. doi: 10.1111/j.1365-3040.2005.01306.x 16021787

[B128] Mansfield T. J. Atkinson C. J. (1990). “Stomatal behaviour in water stressed plants,” in Stress responses in plants: Adaptation and acclimation mechanisms. Eds. Alscher R. G. Cumming J. R. (New York: Wiley-Liss), 241–264.

[B129] Mao P. Zhang Y. Cao B. Guo L. Shao H. Cao Z.. (2016). Effects of salt stress on eco-physiological characteristics in robinia pseudoacacia based on salt-soil rhizosphere. Sci. Total Environ. 568, 118–123. doi: 10.1016/j.scitotenv.2016.06.012 27289394

[B130] Margoshes M. Vallee B. L. (1957). A cadminium protein from equine kidney cortex. J. Am. Chemist Soc. 79, 4813–4814. doi: 10.1021/ja01574a064

[B131] Murkhart A. H. (1985). Comparative water relations of phaseolus vulgaris l. and phaseolus acutinifolius Gray. Plant Physiol. 77, 113–117. doi: 10.1104/pp.77.1.113 16663991 PMC1064467

[B132] Martin-StPaul N. Delzon S. Cochard H. (2017). Plant resistance to drought depend on the timely stomatal closure. Ecol. Lett. 20, 1437–1442. doi: 10.1111/ele.12851 28922708

[B133] Massange-Sánchez J. A. Palmeros-Suárez P. A. Espitia-Rangel E. Rodríguez-Arévalo I. Sánchez-Segura L. Martínez-Gallardo N. A.. (2016). Overexpression of grain amaranth (Amaranthus hypochondriacus) AhERF or AhDOF transcription factors in arabidopsis thaliana increases water deficit- and salt-stress tolerance, respectively, via contrasting stress amelioration mechanisms. PloS One 11, 164280. doi: 10.1371/journal.pone.0164280 PMC5066980 27749893

[B134] Maxwell K. Johnson G. N. (2000). Chlorophyll fluorescence - a practical guide. J. Exp. Bot. 51, 659–668. doi: 10.1093/jexbot/51.345.659 10938857

[B135] McLachlan D. H. Pridgeon A. J. Hetherington A. M. (2018). How arabidopsis talks to itself about its water supply. Mol. Cell 70, 991–992. doi: 10.1016/j.molcel.2018.06.011 29932910

[B136] Mekawy A. A.M. Assaha D. V.M. Munehiro R. Kohnishi E. Nagaoka T. Ueda A.. (2018). Characterization of type 3 metallothionein-like gene (OsMT-3a) from rice, revealed its ability to confer tolerance to salinity and heavy metal stresses. Environmental and Experimental Botany 147, 157–166. doi: 10.1016/j.envexpbot.2017.12.002

[B137] Menezes-Benavente L. Kernodle S. P. Margis-Pinheiro M. Scandalios J. G. (2004). Salt-induced antioxidant metabolism defenses in maize (Zea mays L.) seedlings. Redox Rep. 9, 29–36. doi: 10.1179/135100004225003888 15035825

[B138] Michaletti A. Naghavi M. R. Toorchi M. Zolla L. Rinalducci S. (2018). Metabolomics and proteomics reveal drought-stress responses of leaf tissues from spring-wheat. Sci. Rep. 8, 5710. doi: 10.1038/s41598-018-24012-y 29632386 PMC5890255

[B139] Mishra D. Shekhar S. Singh D. Chakraborty S. Chakraborty N. (2018). “Heat shock proteins and abiotic stress tolerance in plants,” in Regulation of heat shock protein responses. heat shock proteins, vol. 13 . Eds. Asea A. Kaur P. (Cham: Springer).

[B140] Miyake C. Asada K. (1996). Inactivation mechanism of ascorbate peroxidase at low concentrations of ascorbate; hydrogen peroxide decomposes compound I of ascorbate peroxidase. Plant Cell Physiol. 37, 423–430. doi: 10.1093/oxfordjournals.pcp.a028963

[B141] Modarelli G. C. Rouphael Y. De Pascale S. Öztekin G. B. Tüzel Y. Orsini F.. (2020). Appraisal of salt tolerance under greenhouse conditions of a cucurbitaceae genetic repository of potential rootstocks and scions. Agronomy 10, 967. doi: 10.3390/agronomy10070967

[B142] Mo Y. Yang R. Liu L. Gu X. Yang X. Wang Y.. (2015). Growth, photosynthesis and adaptive responses of wild and domesticated watermelon genotypes to drought stress and subsequent re-watering. Plant Growth Regulators 79, 229–241. doi: 10.1007/s10725-015-0128-9

[B143] Mtumtum N. P. (2012). Performance of wild watermelon (Citrullus lanatus l.) in response to population density and mulch (University of Kwazulu-Natal, Pietermaritzburg, RSA: MSc Thesis, School of Agricultural, Earth and Environmental Sciences).

[B144] Munns R. Tester M. (2008). Mechanisms of salinity tolerance. The Annual of Plant Biology. 59, 651–681. doi: 10.1146/annurev.arplant.59.032607.092911 18444910

[B145] Nakaminami K. Okamoto K. Higuchi-Takeuchi M. Yoshizumi T. Yamaguchi Y. Fukao Y.. (2018). AtPep3 is a hormone-like peptide that plays a role in the salinity stress tolerance of plants. Proc. Natl. Acad. Sci. 115, 5810–5815. doi: 10.1073/pnas.1719491115 29760074 PMC5984501

[B146] Nanasato Y. Akashi K. Yokota A. (2015). Co-Expression of cytochrome b561 and ascorbate oxidase in leaves of wild watermelon under drought and high light conditions. Plant Cell Physiol. 46, 1515–1524. doi: 10.1093/pcp/pci164 16020428

[B147] Nanasato Y. Miyake C. Takahara K. Kohzuma K. Munekage Y. N. Yokota A.. (2010). “Mechanisms of drought and high light stress tolerance studied in a xerophyte, citrullus lanatus (wild watermelon),” in The chloroplast. advances in photosynthesis and respiration (Vol. 31). basics and applications. Ed. Rebeiz C. A. (Dordrecht, Netherlands: Springer), 363–377.

[B148] Negi S. Tak H. Ganapathi T. R. (2018). A banana NAC transcription factor (MusaSNAC1) impart drought tolerance by modulating stomatal closure and H2O2 content. Plant Mol. Biol. 96, 457–471. doi: 10.1007/s11103-018-0710-4 29470695

[B149] Neto A. A. Prisco J. T. Eneas-Filho J. Feitosa de Lacerda C. Silva J. V. (2004). Effects of salt stress onplant growth, stomatal response and solute accumulation on different maize genotpes. Brazillian J. Plant Physiol. 16, 31–38. doi: 10.1590/S1677-04202004000100005

[B150] Nguyen N. H. Cheong J. (2018). H2A.Z-containing nucleosomes are evicted to activate AtMYB44 transcription in response to salt stress. Biochem. Biophys. Res. Commun. 499, 1039–1043. doi: 10.1016/j.bbrc.2018.04.048 29649476

[B151] Niinemets Ü. (2015). Uncovering the hidden facets of drought stress: secondary metabolites make the difference. Tree Physiol. 36, 129–132. doi: 10.1093/treephys/tpv128 26687175

[B152] Nilsson J. Weis K. Kjems J. J. (2002). The c-terminal extension of the small GTPase ran is essential for defining the GDP-bound form. Mol. Biol. 318, 583–593. doi: 10.1016/S0022-2836(02)00040-2 12051861

[B153] Niones J. M. Inukai Y. Suralta R. R. Yamauchi A. (2015). QTL associated with lateral root plasticity in response to soil moisture fluctuation stress in rice. Plant Soil 391, 63–75. doi: 10.1007/s11104-015-2404-x

[B154] Niu X. Luo T. Zhao H. Su Y. Ji W. Li H. (2020). Identification of wheat DREB genes and functional characterization of TaDREB3 in response to abiotic stresses. Gene 740, 144514. doi: 10.1016/j.gene.2020.144514 32112985

[B155] Nzengue Y. Steiman R. Rachidi W. Favier A. Guiraud P. (2012). Oxidative stress induced by cadimium in the C6 cell line: role of copper and zinc. Biol. Trace Elements Res. 146, 410–419. doi: 10.1007/s12011-011-9265-9 22127830

[B156] Oh J. E. Kwon Y. Kim J. H. Noh H. Hong S. W. Lee H. (2011). A dual role for MYB60 in stomatal regulation and root growth of arabidopsis thaliana under drought stress. Plant Mol. Biol. 77, 91–103. doi: 10.1007/s11103-011-9796-7 21637967

[B157] Olejnik P. Mądrzak C. J. Nuc K. (2021). Cyclophilins and their functions in abiotic stress and plant–microbe interactions. Biomolecules 11, 1390. doi: 10.3390/biom11091390 34572603 PMC8464771

[B158] Orzechowska A. Trtílek M. Tokarz K. M. Szymańska R. Niewiadomska E. Rozpądek P.. (2021). Thermal analysis of stomatal response under salinity and high light. Int. J. Mol. Sci. 22, 4663. doi: 10.3390/ijms22094663 33925054 PMC8124565

[B159] Pandey S. Fartyal D. Agarwal A. Shukla T. James D. Kaul T.. (2017). Abiotic Stress Tolerance in Plants: Myriad Roles of Ascorbate Peroxidase. Front. Plant Sci. 8, 581. doi: 10.3389/fpls.2017.00581 28473838 PMC5397514

[B160] Park S. I. Kim J. J. Shin S. Y. Kim Y. S. Yoon H. S. (2020). ASR enhances environmental stress tolerance and improves grain yield by modulating stomatal closure in rice. Front. Plant Sci. 10, 1752. doi: 10.3389/fpls.2019.01752 32117337 PMC7033646

[B161] Patankar H. V. Al-Harrasi I. Al Kharusi L. Jana G. A. Al-Yahyai R. Sunkar R.. (2019). Overexpression of a metallothionein 2A gene from date palm confers abiotic stress tolerance to yeast and arabidopsis thaliana. Int. J. Mol. Sci. 20, 2871. doi: 10.3390/ijms20122871 31212812 PMC6627811

[B162] Percival G. C. (2005). The use of chlorophyll fluorescence to identify chemical and environmental stress in leaf tissue of three oak (Quercus) species. J. Arboric 31, 215–227. doi: 10.48044/jauf.2005.028

[B163] Percival G. C. Sheriffs C. N. (2002). Identification of drought-tolerant woody perennials using chlorophyll fluorescence. J. Arboric 28, 215–223.

[B164] Pirasteh-Anosheh H. Saed-Moucheshi A. Pakniyat H. Pessarakli M. (2016). “Stomatal responses to drought stress,” in Water stress and crop plants: a sustainable approach (Chichester: Wiley).

[B165] Priya M. Dhanker O. P. Siddique K. H. M. HanumanthaRao B. Nair R. M. Pandey S.. (2019). Drought and heat stress-related proteins: an update about their functional relevance in imparting stress tolerance in agricultural crops. Theor. Appl. Genet. 132, 1607–1638. doi: 10.1007/s00122-019-03331-2 30941464

[B166] Qu X. Cao B. Kang J. Wang X. Han X. Jiang W.. (2019). Fine-tuning stomatal movement through small signaling peptides. Front. Plant Sci. 10, 69. doi: 10.3389/fpls.2019.00069 30804962 PMC6371031

[B167] Ram C. Danish S. Kesawat M. S. Panwar B. S. Verma M. Arya L.. (2022). Genome-wide comprehensive characterization and expression analysis of TLP gene family revealed its responses to hormonal and abiotic stresses in watermelon (Citrullus lanatus). Gene 844, 146818. doi: 10.1016/j.gene.2022.146818 35985412

[B168] Raturi G. Kumawat S. Mandlik R. Duhan D. Thakral V. Sudhakaran S. (2022). Deciphering the role of aquaporins under different abiotic stress conditions in watermelon (Citrullus lanatus). J. Plant Growth Regulation. doi: 10.1007/s00344-022-10776-1

[B169] Rose A. Meier I. (2001). A domain unique to plant RanGAP is responsible for its targeting to the plant nuclear rim. PNAS 98, 15377–15382. doi: 10.1073/pnas.261459698 11752475 PMC65037

[B170] Rosero A. Granda L. Berdugo-Cely J. A. Šamajová O. Šamaj J. Cerkal R. (2020). A dual strategy of breeding for drought tolerance and introducing drought-tolerant, underutilized crops into production systems to enhance their resilience to water deficiency. Plants (Basel) 9, 1263. doi: 10.3390/plants9101263 32987964 PMC7600178

[B171] Roy R. Agrawal V. Gupta S. C. (2009). Comparison of drought-induced polypeptides and ion leakage in three tomato cultivars. Biol. Plantarum 53, 685–690. doi: 10.1007/s10535-009-0123-y

[B172] Ruibal C. Castro A. Carballo V. Szabados L. Vidal S. (2013). Recovery from heat, salt and osmotic stress in Physcomitrella patens requires a functional small heat shock protein PpHsp16.4. BMC Plant Biol 13, 174. doi: 10.1186/1471-2229-13-174 24188413 PMC4228350

[B173] Rush M. G. Drivas G. D'Eustachio P. (1996). The small nuclear GTPase ran: how much does it run? Bioessays 18, 103–112. doi: 10.1002/bies.950180206 8851043

[B174] Ruttkay-Nedecky B. Nejdl N. Gumulec J. Zitka O. (2013). The role of metallothionein in oxidative stress. Int. J. Mol. Sci. 14, 6044–6066. doi: 10.3390/ijms14036044 23502468 PMC3634463

[B175] Sade N. Moshelion M. (2017). “Plant aquaporins and abiotic stress,” in Plant aquaporins. signaling and communication in plants. Eds. Chaumont F. Tyerman S. (Cham: Springer).

[B176] Sage R. F. McKown A. D. (2006). Is C4 phoptosynthesis less phenotypically plastic than C3 photosynthesis? J. Exp. Bot. 57, 303–317. doi: 10.1093/jxb/erj040 16364950

[B177] Sanda S. Yoshida K. Kuwano M. Kawamura T. Nakajima M. Akashi K.. (2011). Responses of the photosynthetic electron transport system to excess light energy caused by water deficit in wild watermelon. Physiol. Plant 142, 247–264. doi: 10.1111/j.1399-3054.2011.01473.x 21438881

[B178] Scheffzek K. Klebe C. Fritz-Wolf K. Kabsch W. Wittinghofer A. (1995). Crystal structure of the nuclear ras-related protein ran in its GDP-bound form. Nature 374, 378–381. doi: 10.1038/374378a0 7885480

[B179] Schleiff U. Muscolo A. (2011). Fresh look at plant salt tolerance as affected by dynamics at the soil/root-interface using leek and rape as model crops. Eur. J. Plant Sci. Biotechnol. 5, 27–32. doi: 10.13140/2.1.4383.4249

[B180] Seymen M. Yavuz D. Ercan M. Akbulut M. Çoklar H. Kurtar E. S.. (2021). Effect of wild watermelon rootstocks and water stress on chemical properties of watermelon fruit. Horticulture Environment Biotechnol. 62, 411–422. doi: 10.1007/S13580-020-00329-4

[B181] Shalata A. Mittova V. Volokita M. Guy M. Tal M. (2001). Response of the cultivated tomato and its wild salt-tolerant relative Lycopersicon pennellii to salt-dependent oxidative stress: The root antioxidative system. Physiol Plant. 112, 487–494. doi: 10.1034/j.1399-054.2001.1120405.x 11473708

[B182] Shao H. Wang H. Tang X. (2015). NAC transcription factors in plant multiple abiotic stress responses: progress and prospects. Front. Plant Sci. 6, 902. doi: 10.3389/fpls.2015.00902 26579152 PMC4625045

[B183] Sheoran S. Kaur Y. Kumar S. Shukla S. Rakshit S. Kumar R. (2022). Recent advances for drought stress tolerance in maize (Zea mays l.): Present status and future prospects. Front. Plant Sci. 13, 872566. doi: 10.3389/fpls.2022.872566 35707615 PMC9189405

[B184] Shigeoka S. Ishikawa T. Tamoi M. Miyagawa Y. Takeda T. Yabuta Y.. (2002). Regulation and function of the ascorbate peroxidase isoenzymes. J. Exp. Bot. 53, 1305–1319. doi: 10.1093/jexbot/53.372.1305 11997377

[B185] Shikanai T. Takeda T. Yamauchi H. Sano S. Tomizawa K. Yokota A.. (1998). Inhibition of ascorbate peroxidase under oxidative stress in tobacco having bacterial catalase in chloroplasts. FEBS Lett. 428, 47–51. doi: 10.1016/S0014-5793(98)00483-9 9645472

[B186] Simeoni F. Skirycz A. Simoni L. Castorina G. Souza L. P. Fernie A. R.. (2022). The AtMYB60 transcription factor regulates stomatal opening by modulating oxylipin synthesis in guard cells. Sci. Rep. 12 (1), 533. doi: 10.1038/s41598-021-04433-y 35017563 PMC8752683

[B187] Singh B. K. Ramkumar M. K. Dalal M. Singh A. Solanke A. U. Singh N. K.. (2021). Allele mining for a drought responsive gene DRO1determining root growth angle in donors of drought tolerance in rice (Oryza sativa l.). Physiol. Mol. Biol. Plants 27, 523–534. doi: 10.1007/s12298-021-00950-2 33854281 PMC7981370

[B188] Singh S. K. Reddy K. R. (2011). Regulation of photosynthesis, fluorescence, stomatal conductance and water-use efficiency of cowpea (Vigna unguiculata [L.] walp.) under drought. J. Photochem. Photobiol. 105, 40–50. doi: 10.1016/j.jphotobiol.2011.07.001 21820316

[B189] Si Y. Zhang C. Meng S. Dane F. (2009). Gene expression changes in response to drought stress in citrullus colocynthis. Plant Cell Rep. 28, 997–1009. doi: 10.1007/s00299-009-0703-5 19415285

[B190] Sminorff N. (1993). The role of active oxygen in the response of plants to water deficit and desiccation. New Phytol 125, 27–58. doi: 10.1111/j.1469-8137.1993.tb03863.x 33874604

[B191] Smith B. (2006). The farming book (Pietermaritzburg: University of Kwazulu-Natal Press).

[B192] Smith S. Zhu S. Joos L. Roberts I. Nikonorova N. Vu L. D.. (2020). The CEP5 peptide promotes abiotic stress tolerance, as revealed by quantitative proteomics, and attenuates the AUX/IAA equilibrium in arabidopsis. Mol. Cell Proteomics 19, 1248–1262. doi: 10.1074/mcp.RA119.001826 32404488 PMC8011570

[B193] Soda N. Sharan A. Gupta B. Singla-Pareek S. L. Pareek A. (2016). Evidence for nuclear interaction of a cytoskeleton protein (OsIFL) with metallothionein and its role in salinity stress tolerance. Sci Rep 6, 34762. doi: 10.1038/srep34762 27708383 PMC5052524

[B194] Sofo A. Scopa A. Nuzzaci M. Vitti A. (2015). Ascorbate Peroxidase and Catalase Activities and Their Genetic Regulation in Plants Subjected to Drought and Salinity Stresses. Int J Mol Sci. 16, 13561–13578. doi: 10.3390/ijms160613561 26075872 PMC4490509

[B195] Song X. F. Guo P. Ren S. C. Xu T. T. Liu C. M. (2013). Antigonistic peptide technology for functional dissection of CLV3/ESR genes in arabidopsis. Plant Cell Physiol. 161, 1076–1085. doi: 10.1104/pp.112.211029 PMC3585580 23321419

[B196] Song Q. Joshi M. DiPiazza J. Joshi V. (2020). Functional relevance of citrulline in the vegetative tissues of watermelon during abiotic stresses. Front. Plant Sci. 11, 512. doi: 10.3389/fpls.2020.00512 32431723 PMC7216109

[B197] Song Q. Joshi M. Joshi V. (2020). Transcriptomic analysis of short-term salt stress response in watermelon seedlings. Int. J. Mol. Sci. 21, 6036. doi: 10.3390/ijms21176036 32839408 PMC7504276

[B198] Stepien P. Johnson G. N. (2009). Contrasting responses of photosynthesis to salt stress in the glycophyte Arabidopsis and the halophyte thellungiella: role of the plastid terminal oxidase as an alternative electron sink. Plant Physiol. 149, 1154–65. doi: 10.1104/pp.108.132407 19052149 PMC2633845

[B199] Sui D. Wang B. (2020). Transcriptome analysis reveals complex defensive mechanisms in salt-tolerant and salt-sensetive shrub willow genotypes under salinity stress. International journal of genomics. 6870157. doi: 10.1155/2020/6870157 32775403 PMC7407064

[B200] Sun J. Cao H. Cheng J. He X. Sohail H. Niu M.. (2018). Pumpkin CmHKT1;1 controls shoot na+ accumulation *via* limiting na+ transport from rootstock to scion in grafted cucumber. Int. J. Mol. Sci. 19, 2648. doi: 10.3390/ijms19092648 30200653 PMC6165489

[B201] Sussmilch F. C. Brodribb T. J. McAdam S. A. M. (2017). Up-regulation of NCED3 and ABA biosynthesis occur within minutes of a decrease in leaf turgor but AHK1 is not required. J. Exp. Bot. 68, 2913–2918. doi: 10.1093/jxb/erx124 28449122 PMC5853609

[B202] Takahashi F. Kuromori T. Urano K. Yamaguchi-Shinozaki K. Shinozaki K. (2020). Drought stress responses and resistance in plants: From cellular responses to long- distance intercellular communication. Front. Plant Sci. 11, 556972. doi: 10.3389/fpls.2020.556972 33013974 PMC7511591

[B203] Takahashi F. Suzuki T. Osakabe U. Betsuyaku S. Kondo Y. Dohmae N.. (2018). A small peptide modulates stomatal control *via* abscisic acid in long-distance signaling. Nature 556, 235–238. doi: 10.1038/s41586-018-0009-2 29618812

[B204] Tan C. Qiao H. Ma M. Wang X. Tian Y. Bai S.. (2021). Genome-wide identification and characterization of melon bHLH transcription factors in regulation of fruit development. Plants 10, 2721. doi: 10.3390/plants10122721 34961193 PMC8709311

[B205] Tátrai Z. A. Sanoubar R. Pluhár Z. Mancarella S. Orsini F. Gianquinto G. (2016). Morphological and Physiological Plant Responses to Drought Stress in Thymus citriodorus. International Journal of Agronomy 2016, 4165750. doi: 10.1155/2016/4165750

[B206] Thao N. P. Thu N. B. A. Hoang X. L. T. Ha C. V. Tran L. S. P. (2013). Differential expression analysis of a subset of drought-responsive GmNAC genes in two soybean cultivars differing in drought tolerance. Int. J. Mol. Sci. 14, 23828–23841. doi: 10.3390/ijms141223828 24322442 PMC3876080

[B207] Tian F. Hu X. L. Yao T. Yang X. Chen J. G. Lu M. Z. (2021). Recent Advances in the Roles of HSFs and HSPs in Heat Stress Response in Woody Plants. Front. Plant Sci. 12, 704905. doi: 10.3389/fpls.2021.704905 34305991 PMC8299100

[B208] Tissue D. T. Griffin K. L. Turnbull M. H. Whitehead D. (2005). Stomatal and non-stomatal limitations to photosynthesis in four tree species in a temperate rainforest dominated by dacrydium cupressinum in new Zealand. Tree Physiol. 25, 447–456. doi: 10.1093/treephys/25.4.447 15687093

[B209] Tiwari P. Chakrabarty D. (2021). Dehydrin in the past four decades: From chaperones to transcription co-regulators in regulating abiotic stress response. Curr. Res. Biotechnol. 3, 249–259. doi: 10.1016/j.crbiot.2021.07.005

[B210] Tsuji W. Ali M. Inanaga S.. (2013). Growth and gas exchange of three sorghum cultivars under drought stress. Biol. Plantarum 46, 583–587. doi: 10.1023/A:1024875814296

[B211] Uga Y. Sugimoto K. Ogawa S. Rane J. Ishitani M. Hara N.. (2013). Control of root system architecture by DEEPER ROOTING 1 increases rice yield under drought conditions. Nat. Genet. 45, 1097–1102. doi: 10.1038/ng.2725 23913002

[B212] Urbano-Gámez J. A. El-Azaz J. Ávila C. de la Torre F. N. Cánovas F. M. (2020). Enzymes involved in the biosynthesis of arginine from ornithine in maritime pine (Pinus pinaster ait.). Plants (Basel) 9, 1271. doi: 10.3390/plants9101271 32992504 PMC7601404

[B213] Vandeleur R. K. Mayo G. Shelden M. C. Gilliham M. Kaiser B. N. Tyerman S. D. (2009). The role of plasma membrane intrinsic protein aquaporins in water transport through roots: diurnal and drought stress responses reveal different strategies between isohydric and anisohydric cultivars of grapevine. Plant Physiol. 149, 445–460. doi: 10.1104/pp.108.128645 18987216 PMC2613730

[B214] Vandeleur R. K. Sullivan W. Athman A. Jordans C. Gilliham M. Kaiser B. N.. (2014). Rapid shoot-to-root signalling regulates root hydraulic conductance *via* aquaporins. Plant Cell Environ. 37, 520–538. doi: 10.1111/pce.12175 23926961

[B215] Vera-Estrella R. (2004). Novel regulation of aquaporins during osmotic stress. Plant Physiol. 135, 2318–2329. doi: 10.1104/pp.104.044891 15299122 PMC520800

[B216] Verslues P. E. Juenger T. E. (2011). Drought, metabolites, and arabdopsis natural variaton: a promising combination for understanding adaptation to water-limited environments. Curr. Opin. Plant Biol. 14, 240–245. doi: 10.1016/j.pbi.2011.04.006 21561798

[B217] Walter J. Nagy L. Hein R. Rasscher U. Beikerkuhnlein C. Willner E.. (2011). Do plants remember drought? hints towards a drought-memory in grasses. Environ. Exp. Bot. 71, 34–40. doi: 10.1016/j.envexpbot.2010.10.020

[B218] Wang A. Guo J. Wang S. Zhang Y. Lu F. Duan J.. (2022). BoPEP4, a c-terminally encoded plant elicitor peptide from broccoli, plays a role in salinity stress tolerance. Int. J. Mol. Sci. 23, 3090. doi: 10.3390/ijms23063090 35328511 PMC8952307

[B219] Wang W. Liu Z. Bao L. J. Zhang S. S. Zhang C. G. Li X.. (2017). The RopGEF2-ROP7/ROP2 pathway activated by phyB suppresses red light-induced stomatal opening. Plant Physiol. 174, 717–731. doi: 10.1104/pp.16.01727 28188273 PMC5462004

[B220] Wang X. Niu Y. Zheng Y. (2021). Multiple functions of MYB transcription factors in abiotic stress responses. Int. J. Mol. Sci. 22, 6125. doi: 10.3390/ijms22116125 34200125 PMC8201141

[B221] Wang X. Xu Y. Han Y. Bao S. Du J. Yuan M.. (2006). Overexpression of RAN1 in rice and arabidopsis alters primodial meristem, mitotics progress and sensitivity to auxin. Plant Physiol. 140, 91–101. doi: 10.1104/pp.105.071670 16361516 PMC1326034

[B222] Wang L. M. Zhang L. D. Chen J. B. Huang D. F. Zhang Y. D. (2016). Physiological analysis and transcriptome comparison of two muskmelon (Cucumis melo l.) cultivars in response to salt stress. Genet. Mol. Res. 15, gmr.15038738. 10.4238/gmr.15038738 27706747

[B223] Wang D. Zhichen Cao Z. Wang W. Zhu W. Xiaocong Hao X. Fang Z.. (2020). Genome-wide characterization of OFP family genes in wheat (Triticum aestivum l.) reveals that TaOPF29a-a promotes drought tolerance. BioMed. Res. Int. 2020, 9708324. doi: 10.1155/2020/9708324 33224986 PMC7666709

[B224] Wan Q. Luo L. Zhang X. Lv Y.m Zhu S. Kong L. Wan Y.. (2021). Genome-wide identification and abiotic stress response pattern analysis of NF-y gene family in peanut (Arachis hypogaea l.). Trop. Plant Biol. 14, 329–344. doi: 10.1007/s12042-021-09295-2

[B225] Wang Y. Wisniewski M. Meilan R. Cui M. Webb R. Fuchigami L. (2005). Overexpression of cytosolic ascorbate peroxidase in tomato (Lycopersicon esculentumin tomato (Lycopersicon esculentumin tomato ( L.) confers tolerance to chilling and salt stress. J.J.J ASHS 130, 167–173.

[B226] Wasaya A. Zhang X. Fang Q. Yan Z. (2018). Root phenotyping for drought tolerance: A review. Agronomy 8, 241. doi: 10.3390/agronomy8110241

[B227] Weisany W. Sohrabi Y. Heidari G. Siosemardeh A. Ghassemi-Golezani K. (2021). Changes in antioxidant enzymes activity and plant performance by salinity stress and zinc application in soybean (Glycine max L.). Plant. Omics. 5, 60–67.

[B228] Xu P. Cai W. (2014). RAN1 is involved in plant cold resistance and development in rice (Oryza sativa). J. Exp. Bot. 65, 3277–3287. doi: 10.1093/jxb/eru178 24790113 PMC4071843

[B229] Xu Q. He J. Dong J. Hou X. Zhang X. (2018). Genomic survey and expression profiling of the MYB gene family in watermelon. Hortic. Plant J. 4, 1–15. doi: 10.1016/j.hpj.2017.12.001

[B230] Xu P. Zang A. Chen H. Cai W. (2016). The small G protein AtRAN1 regulates vegetative growth and stress tolerance in arabidopsis thaliana. PloS One 11, e0154787. doi: 10.1371/journal.pone.0154787 27258048 PMC4892486

[B231] Yaginuma H. Hirakawa Y. kondo Y. Ohashi-Ito K. Fukuda H. (2011). A novel function of the TDIF-related peptides: promotion of axillary bud formation. Plant Cell Physiol. 52, 1354–1364. doi: 10.1093/pcp/pcr081 21693505

[B232] Yamori W. Hikosaka K. Way D. A. (2014). Temperature response of photosynthesis in C3, C4 and CAM plants: temperature acclimation and temperature adaptation. Photosynthesis Res. 119, 101–117. doi: 10.1007/s11120-013-9874-6 23801171

[B233] Yang Z. (2002). Small GTPases: versatile signalling switches in plants. Plant Cell 14, S375–S388. doi: 10.1105/tpc.001065 12045289 PMC151267

[B234] Yang H. Hu J. X. Long X. H. Liu Z. P. Rengel Z. (2016). Salinity altered root distribution and increased diversity of bacterial communities in the rhizosphere soil of Jerusalem artichoke. Sci. Rep. 6, 20687. doi: 10.1038/srep20687 26852800 PMC4745076

[B235] Yang X. Li H. Yang Y. Wang Y. Mo Y. Zhang R. (2018). Identification and expression analyses of WRKY genes reveal their involvement in growth and abiotic stress response in watermelon (Citrullus lanatus). PloS One 13, e0191308. doi: 10.1371/journal.pone.0191308 29338040 PMC5770075

[B236] Yang Y. Mo Y. Yang X. Zhang H. Wang Y. Li H.. (2016). Transcriptome profiling of watermelon root in response to short-term osmotic stress. PloS One 11 (11), e0166314. doi: 10.1371/journal.pone.0166314 27861528 PMC5115733

[B237] Yang Y. Wang L. Tian J. Li J. Sun J. He L.. (2012). Proteomic study participating the enhancement of growth and salt tolerance of bottle gourd rootstock-grafted watermelon seedlings. Plant Physiol. Biochem. 58, 54–65. doi: 10.1016/j.plaphy.2012.05.026 22771436

[B238] Yang S. Xu K. Chen S. Li T. Xia H. Chen L.. (2019). A stress-responsive bZIP transcription factor OsbZIP62 improves drought and oxidative tolerance in rice. BMC Plant Biol. 19, 260. doi: 10.1186/s12870-019-1872-1 31208338 PMC6580479

[B239] Yang J. Zhu J. Yang Y. (2017). Genome-wide identification and expression analysis of NF-y transcription factor families in watermelon (Citrullus lanatus). J. Plant Growth Regulators 36, 590–607. doi: 10.1007/s00344-017-9670-1

[B240] Yang Y. Wang B. Wang J. He C. Zhang D. Li P.. (2022). Transcription factors ZmNF-YA1 and ZmNF-YB16 regulate plant growth and drought tolerance in maize. Plant Physiol. 190, 1506–1525. doi: 10.1093/plphys/kiac340 35861438 PMC9516732

[B241] Yan Y. Wang S. Wei M. Gong B. Shi Q. (2018). Effect of different rootstocks on the salt stress tolerance in watermelon seedlings. Hortic. Plant J. 4, 239–249. doi: 10.1016/j.hpj.2018.08.003

[B242] Yan W. Zheng S. Zhong Y. Shangguan Z. (2017). Contrasting dynamics of leaf potential and gas exchange during progressive drought cycles and recovery in amorpha fruticosa and robinia pseudoacacia. Sci. Rep. 7, 4470. doi: 10.1038/s41598-017-04760-z 28667337 PMC5493649

[B243] Yildiz M. Poyraz İ. Çavdar A. Özgen Y. Beyaz R. (2020). Plant Responses to Salt Stress. In (Ed.), Plant Breeding - Current and Future Views. IntechOpen. doi: 10.5772/intechopen.93920

[B244] Yokota A. Kawasaki S. Iwano M. Nakamura C. Miyake C. Akashi K. (2002). Citrulline and DRIP-1 protein (ArgE homologue) in drought tolerance of wild watermelon. Ann. Bot. 89, 825–832. doi: 10.1093/aob/mcf074 12102508 PMC4233801

[B245] Yoshimura K. Ishikawa T. Nakamura Y. Tamoi M. Takeda T. Tada T.. (1998). Comparative study on recombinant chloroplastic and cytosolic ascorbate peroxidase isozymes of spinach. Arch. Biochem. Biophys. 353, 55–63. doi: 10.1006/abbi.1997.0612 9578600

[B246] Yoshimura K. Masuda A. Kuwano M. Yokota A. Akashi K. (2008). Programmed proteome response for drought avoidance/tolerance in the root of a C3 xerophyte (wild watermelon) under water deficits. Plant Cell Physiol. 49, 226–241. doi: 10.1093/pcp/pcm180 18178965

[B247] Yuan G. Liu J. An G. Li W. Si W. Sun D.. (2022a). Genome-wide identification and characterization of the trehalose-6-Phosphate synthetase (TPS) gene family in watermelon (Citrullus lanatus) and their transcriptional responses to salt stress. Int. J. Mol. Sci. 23, 276. doi: 10.3390/ijms23010276 PMC8745194 35008702

[B248] Yuan G. Sun D. An G. Li W. Si W. Liu J.. (2022b). Transcriptomic and metabolomic analysis of the effects of exogenous trehalose on salt tolerance in watermelon (Citrullus lanatus). Cells 11, 2338. doi: 10.3390/cells11152338 35954182 PMC9367363

[B249] Yu C. Yan M. Dong H. Luo J. Ke Y. Guo A.. (2021). Maize bHLH55 functions positively in salt tolerance through modulation of AsA biosynthesis by directly regulating GDP-mannose pathway genes. Plant Sci. 302, 110676. doi: 10.1016/j.plantsci.2020.110676 33288001

[B250] Zakhidov E. Nematov S. Kuvondikov V. (2016). “Monitoring of the drought tolerance of various cotton genotypes using chlorophyll fluorescence,” in Applied photosynthesis. Ed. Najafpour M. M. London: IntechOpen Limited.

[B251] Zarei S. Ehsanpour A. A. Abbaspour J. (2012). The role of over-expression of P5CS gene on proline, catalase, ascorbate peroxidase activity and lipid peroxidation of transgenic tobacco (Nicotiana tabacum l.) plant under *in vitro* drought stress. J. Cell. Mol. Res. 4, 43–49. doi: 10.22067/jcmr.v4i1.18249

[B252] Zhang X. Chen L. Shi Q. Ren Z. (2020). SlMYB102, an R2R3-type MYB gene, confers salt tolerance in transgenic tomato. Plant Sci. 291, 110356. doi: 10.1016/j.plantsci.2019.110356 31928668

[B253] Zhang X. Liu S. Takano T. (2008). Overexpression of a mitochondrial ATP synthase small subunit gene (AtMtATP6) confers tolerance to several abiotic stresses in saccharomyces cerevisiae and arabidopsis thaliana. Biotechnol. Lett. 30, 1289–1294. doi: 10.1007/s10529-008-9685-6 18338219

[B254] Zhang L. Shi X. Zhang Y. Wang J. Yang J. Ishida T.. (2019). CLE9 peptide-induced stomatal closure is mediated by abscisic acid, hydrogen peroxide, and nitric oxide in arabidopsis thaliana. Plant Cell Environ. 42, 1033–1044. doi: 10.1111/pce.13475 30378140

[B255] Zhang G. Zhang M. Zhao Z. Ren Y. Li Q. Wang W. (2017b). Wheat TaPUB1 modulates plant drought stress resistance by improving antioxidant capability. Sci. Rep. 7, 7549. doi: 10.1038/s41598-017-08181-w 28790447 PMC5548723

[B256] Zhang H. Zhao Y. Zhou D. X. (2017a). Rice NAD+-dependent histone deacetylase OsSRT1 represses glycolysis and regulates the moonlighting function of GAPDH as a transcriptional activator of glycolytic genes. Nucleic Acids Res. 45, 12241–12255. doi: 10.1093/nar/gkx825 28981755 PMC5716216

[B257] Zhao P. Hou S. Guo X. Jia J. Yang W. Liu Z.. (2019). A MYB-related transcription factor from sheepgrass, LcMYB2, promotes seed germination and root growth under drought stress. BMC Plant Biol. 19, 564. doi: 10.1186/s12870-019-2159-2 31852429 PMC6921572

[B258] Zhao H. Liang H. Chu Y. Sun C. Wei N. Yang M.. (2019). Effects of salt stress on chlorophyll fluorescence and the antioxidant system in gimkgo biloba l. Seedlings. Hortic. Sci. 54, 2125–2133. doi: 10.21273/HORTSCI14432-19

[B259] Zhou H. Xiao F. Zheng Y. Liu G. Zhuang Y. Wang Z.. (2022). PAMP-INDUCED SECRETED PEPTIDE 3 modulates salt tolerance through RECEPTOR-LIKE KINASE 7 in plants. Plant Cell 34, 927–944. doi: 10.1093/plcell/koab292 34865139 PMC8824610

[B260] Zhu J. Ingram P. A. Benfey P. N. Elich T. (2011). From lab to field, new approaches to phenotyping root system architecture. Curr. Opin. Plant Biol. 14, 310–317. doi: 10.1016/j.pbi.2011.03.020 21530367

[B261] Zhu Y. Yuan G. Gao B. An G. Li W. Si W.. (2022). Comparative transcriptome profiling provides insights into plant salt tolerance in watermelon (Citrullus lanatus). Life 12, 1033. doi: 10.3390/life12071033 35888121 PMC9320501

[B262] Zhu H. Zhao S. Lu X. He N. Gao L. Dou J.. (2018). Genome duplication improves the resistance of watermelon root to salt stress. Plant Physiol. Biochem. 133, 11–21. doi: 10.1016/j.plaphy.2018.10.019 30384081

[B263] Zivcak M. Kalaji H. M. Shao H. B. Olsovska K. Brestic M. (2014). Photosynthetic proton and electron transport in wheat leaves under prolonged moderate drought stress. J. Photochem. Photobiol. 137, 107–115. doi: 10.1016/j.jphotobiol.2014.01.007 24508481

[B264] Zulu N. S. Modi A. T. (2010). A preliminary study to determine water stress tolerance in wild melon (Citrulus lanatus l.). South Afr. J. Plant Soil 27, 334–336. doi: 10.1080/02571862.2010.10640004

[B265] Zou J. Liu C. Liu A. Zou D. Chen X. (2021). Overexpression of OsHsp17.0 and OsHsp23.7 enhances drought and salt tolerance in rice. J Plant Physiol. 2169, 628–35. doi: 10.1016/j.jplph.2011.12.014 22321692

